# Calcium dynamics and associated temporal patterns of milk constituents in early-lactation multiparous Holsteins

**DOI:** 10.3168/jds.2022-23142

**Published:** 2023-05-18

**Authors:** J. A. Seminara, K. R. Callero, I. R. Frost, R. M. Martinez, H. A. McCray, A. M. Reid, C. R. Seely, D. M. Barbano, J. A. A. McArt

**Affiliations:** 1 Department of Population Medicine and Diagnostic Sciences, College of Veterinary Medicine, Cornell University, Ithaca, NY 14853; 2 College of Agriculture and Life Sciences, Cornell University, Ithaca, NY 14853; 3 College of Veterinary Medicine, Cornell University, Ithaca, NY 14853; 4 College of Arts and Sciences, Cornell University, Ithaca, NY 14853; 5 Department of Food Science, College of Agriculture and Life Sciences, Cornell University, Ithaca, NY 14853

**Keywords:** subclinical hypocalcemia, Fourier-transform infrared spectroscopy, calcium, fatty acids, milk

## Abstract

At the onset of lactation, calcium (Ca) homeostasis is challenged. For the transitioning dairy cow, inadequate responses to this challenge may result in subclinical hypocalcemia at some point in the postpartum period. It has been proposed that dynamics of blood Ca and the timing of subclinical hypocalcemia allow cows to be classified into 4 Ca dynamic groups by assessing serum total Ca concentrations (tCa) at 1 and 4 days in milk (DIM). These differing dynamics are associated with different risks of adverse health events and suboptimal production. Our prospective cohort study aimed to characterize the temporal patterns of milk constituents in cows with differing Ca dynamics to investigate the potential of Fourier-transform infrared spectroscopic (FTIR) analysis of milk as a diagnostic tool for identifying cows with unfavorable Ca dynamics. We sampled the blood of 343 multiparous Holsteins on a single dairy in Cayuga County, New York, at 1 and 4 DIM and classified these cows into Ca dynamic groups using threshold concentrations of tCa (1 DIM: tCa <1.98 mmol/L; 4 DIM: tCa <2.22 mmol/L) derived from receiver operating characteristic curve analysis based on epidemiologically relevant health and production outcomes. We also collected proportional milk samples from each of these cows from 3 to 10 DIM for FTIR analysis of milk constituents. Through this analysis we estimated the milk constituent levels of anhydrous lactose (g/100 g of milk and g/milking), true protein (g/100 g of milk and g/milking), fat (g/100 g of milk and g/milking), milk urea nitrogen (mg/100 g of milk), fatty acid (FA) groups including de novo, mixed origin, and preformed FA measured in grams/100 g of milk, by relative percentage, and grams/milking, as well as energy-related metabolites including ketone bodies and milk-predicted blood nonesterified FA. Individual milk constituents were compared among groups at each time point and over the entire sample period using linear regression models. Overall, we found differences among the constituent profiles of Ca dynamic groups at approximately every time point and over the entire sample period. The 2 at-risk groups of cows did not differ from each other at more than one time point for any constituent, however prominent differences existed between the milk of normocalcemic cows and the milk of the other Ca dynamic groups with respect to FA. Over the entire sample period, lactose and protein yield (g/milking) were lower in the milk of at-risk cows than in the milk of the other Ca dynamic groups. In addition, milk yield per milking followed patterns consistent with previous Ca dynamic group research. Though our use of a single farm does limit the general applicability of these findings, our conclusions provide evidence that FTIR may be a useful method for discriminating between cows with different Ca dynamics at time points that may be relevant in the optimization of management or development of clinical intervention strategies.

## INTRODUCTION

Immediately following parturition, the transitioning dairy cow experiences a variety of metabolic challenges associated with supporting milk production (Bauman and Currie, 1980). Of these challenges, maintaining calcium (Ca) homeostasis is of central importance due to the extensive roles that Ca plays in normal physiology. Milk production causes the mammary gland to become a sink for Ca to the extent that physiological demand for Ca surpasses the cow’s ability to compensate via DMI alone (Ramberg et al., 1984; Goff et al., 2002). As a result, many multiparous dairy cows will become hypocalcemic at some point during early lactation (Horst et al., 1997; Goff, 2008).

Though clinical hypocalcemia, also known as milk fever or parturient paresis, has historically been the disease of concern, due to prepartum diet management, only 5% of cows will be affected by this disease state (Goff, 2008). Subclinical hypocalcemia (**SCH**) is currently of great relevance as approximately 50% of multiparous dairy cows experience this disease (Reinhardt et al., 2011; Chamberlin et al., 2013), and those that do are at elevated risk for a variety of other adverse health outcomes as well as decreased production and reproductive success (Martinez et al., 2012; Chapinal et al., 2012; McArt and Neves, 2020).

Despite a large body of literature concerning SCH and early-lactation Ca homeostasis, some key details remain contested. Differences in study design have made it difficult for researchers to agree on the threshold level of total serum Ca concentration (**tCa**) used to diagnose SCH or the time at which a diagnostic sample should be collected (Couto Serrenho et al., 2021). Ongoing research has suggested that repeated measurements of tCa may be required to fully understand the dynamics of Ca homeostasis in early-lactation cows (Caixeta et al., 2017; Neves et al., 2018; Venjakob et al., 2021). This work has revealed that measurements of tCa at 1 and 4 DIM can be used to classify cows into 1 of 4 Ca dynamic groups: persistent SCH (**pSCH**; SCH at 1 and 4 DIM), delayed SCH (**dSCH**; SCH at 4 DIM only), transient SCH (**tSCH**; SCH at 1 DIM only) and normocalcemic (**NC**; never SCH). Importantly, pSCH and dSCH cows have been shown to be at a greater risk of adverse health events and suboptimal production than their tSCH and NC counterparts (McArt and Neves, 2020). These findings suggest that tCa must be measured at multiple time points to accurately identify animals that are at increased risk of disease.

Practically, a diagnostic approach involving repeated measures of tCa may not be a viable management strategy, due to the costs and labor involved. Fourier-transform infrared spectroscopic (**FTIR**) analysis of milk presents a potential solution to this problem. Studies have examined FTIR methods for the diagnosis of hyperketonemia and have found associations between milk constituents and early-lactation disease, culling, and production outcomes (Bach et al., 2019; Ho et al., 2021). Relative percentages (**rel%**) of certain fatty acid groups, milk ketone bodies, and milk-predicted blood nonesterified fatty acids (**mpbNEFA**) have all been associated with health status of cows (Jorjong et al., 2015; Luke et al., 2019; Bach et al., 2019). Although these methods show great promise as a herd health monitoring tool, much of the milk FTIR literature is based on data from monthly milk samples and therefore lacks a level of resolution that might be necessary for detection of illness and assessment of individual risk at the cow level (Bach et al., 2019).

Therefore, our objective was to characterize the temporal patterns of milk constituents from 3 to 10 DIM for cows with different Ca dynamics to investigate the potential of FTIR milk constituent analysis as a diagnostic tool with clinical utility. Our secondary aim was to validate previously proposed models of early-postpartum Ca dynamics. We hypothesized that epidemiologically relevant data on health and production outcomes would allow us to classify cows into Ca dynamic groups, and that patterns of milk constituents would be different among these groups. Further, we hypothesized that Ca dynamic groups would differ with respect to their FA profiles as well as the profiles of mpbNEFA and ketone bodies, but they would not differ with respect to lactose or protein. We additionally hypothesized that these differences would be present as early as 3 and 4 DIM, time points at which a successful clinical intervention might be possible.

## MATERIALS AND METHODS

Our animal use protocol was approved by the Cornell University Institutional Animal Care and Use Committee, protocol number 2020–0102. We conducted a prospective cohort study which was analyzed and written following the STROBE-Vet reporting guidelines (Sargeant et al., 2016).

### Study Population

Data were collected from one dairy farm in Cayuga County, New York, from June through July 2021; this farm was selected based on its longstanding history with Cornell’s Ambulatory and Production Medicine Clinic. The farm milked 4,400 Holstein cows 3 times daily and averaged 42.3 kg of milk per cow per day. Cows were housed in freestall barns with concrete floors, bedded with recycled manure solids. Diets fed during the prepartum period were formulated to contain a dietary cation-anion difference of −10 mEq/100 g DM, in accordance with common parturient paresis prevention protocols (Goff, 2008). A total mixed ration formulated for early-lactation cows was fed until cows were moved from the early-lactation pen to a high-production pen or to a sick pen. The formulated early-lactation diet ingredient and nutrient composition is included in Supplemental Table S1 (https://blogs.cornell.edu/jessmcartlab/research/supp/ca-milk/tab-1/) and has been reported elsewhere (Callero et al., 2023). The mean day of pen move from the early-lactation to the high-production or sick pen was 12 DIM but ranged from 3 to 22 DIM.

### Animal Sampling and Analysis

All multiparous Holstein cows that calved during the study period were eligible for enrollment at 1 DIM. Blood samples were collected at 1 and 4 DIM from the coccygeal vessels at 0730 h following morning feed delivery. Samples were collected into 10-mL evacuated tubes without anticoagulant using 20-gauge Vacutainer needles (Becton Dickinson, Franklin Lakes, NJ) and allowed to clot for at least 30 min at room temperature. Serum was separated via centrifugation at 2,000 × *g* for 15 min at 20°C, aliquoted into 1.7-mL microfuge tubes, and stored at −20°C until analysis.

Milk samples were collected during the midday milking at 1030 h, Monday through Friday, on all enrolled cows housed in the early-lactation pen. Cows were milked on a 100-cow rotary parlor (DeLaval, Tumba, Sweden), and proportional milk samples were collected using milk samplers (DeLaval Fat Sampler MM25–27 BC; DeLaval). Samples were mixed 3 times, poured into 60 mL preservative-free plastic sample vials (Aptar CSP Technologies, Auburn, AL), and placed in an ice bath at 4°C until analysis. Cow identification was performed visually during sampling on the parlor floor and verified using parlor software (DelPro; DeLaval). Cows were sampled for the duration of their stay in the early-lactation pen.

Production yield data for each milking was collected daily from the parlor software. Diseases were diagnosed and recorded by on-farm personnel using standard disease definition protocols following physical examination. Cows were flagged for early-lactation disease screening by a farm-based algorithm following a drop in milk production or an otherwise abnormal milking. Cows were diagnosed with hyperketonemia if their cow-side BHB meter blood test reported a BHB concentration ≥1.2 mmol/L. If the classical “ping” sound was detected during simultaneous auscultation and percussion in a line from the tuber coxae to the olecranon, the cow was diagnosed with a displaced abomasum. Cows showing systemic signs of illness with rectal temperatures ≥39.5°C and a red to brown uterine discharge were diagnosed with metritis. Mastitis diagnosis was based on the visual identification of a hot and swollen quarter and presence of abnormal milk with or without garget. Disease data for enrolled cows were extracted from herd management software (DairyComp 305, VAS, Tulare, CA) on a weekly basis. Farm employees were blinded to our study’s purpose throughout the length of the trial.

### Sample Size Calculation and Exclusion Criteria

To determine our enrollment goal, we conducted an a priori power calculation for comparing means for independent samples using Stata v17 (College Station, TX). Based on data reported in Bach et al. (2019), we assumed that cows remaining healthy during the transition period would have a mean de novo FA rel% of 23.8 ± 3.3% whereas those that go on to develop disease would have a mean of 22.0 ± 3.3%. Using these means and standard deviations, controlling for type I error at 5% and setting power at 90%, our power calculation resulted in a required sample size of 72 cows per Ca dynamic group. Assuming that pSCH and dSCH would make up 25% of the study population each (McArt and Neves, 2020; Seely et al., 2021), a total sample size of 288 multiparous cows was required for the study. We additionally inflated our sample size by 10% based on the assumption that some proportion of cows would receive Ca supplementation or have a disease event of interest before 4 DIM. Given the aim of classifying cows into Ca dynamic groups at a clinically useful time point when therapeutic intervention strategies might still be effective, the sample size was inflated by an additional 20% to account for the loss of cows whose 3 and 4 DIM milk samples would not be collected on the weekends. This provided a final enrollment aim of 380 cows.

We excluded cows from analysis for the following reasons: previous days carried calf <260, diagnosis of one of the diseases of interest before 4 DIM, missing tCa data at either 1 or 4 DIM, if they were moved from the early-lactation pen before 3 DIM, or if they did not have valid milk data for any day between 3 and 10 DIM. Individual milk samples were excluded from analysis for the following reasons: if they were collected from cows that had been excluded for the reasons noted above, if they were collected before 3 DIM or after 10 DIM, or if their FTIR data could not be validated. Daily milk FTIR data underwent validation in which milk samples where the sum of de novo, mixed, and preformed FA (g/100 g of milk) was >99% of the total fat concentration (g/100 g of milk) were removed. Samples with summed FA data within the 98 to 99% range that also had negative milk-predicted BHB values were also removed. All samples with a FA sum <97% were retained.

### Sample Analysis

Serum samples were analyzed at the New York State Animal Health Diagnostic Center (Ithaca, NY) on an automated analyzer (Hitachi Modular P800, Roche Diagnostics, Indianapolis, IN) for tCa using commercially available kits (Ca Gen.2, Roche Diagnostics). Inter- and intra-assay coefficients of variation were 1.2 and 0.8%, respectively.

Composition analysis of milk samples was performed daily in the Barbano Laboratory at the Department of Food Science at Cornell University (Ithaca, NY) using an FTIR spectrophotometer (Lactoscope model Combi 600, Delta Instruments, Drachten, the Netherlands) to determine the content (percent by weight) of milk fat, true protein and anhydrous lactose. Prediction models previously described in Kaylegian et al. (2009) for fat, true protein, and anhydrous lactose were the optimized basic model filter wavelengths. Reference methods used to calibrate these models were described by Wojciechowski et al. (2016).

Partial least squares prediction models were used to estimate the grams/100 g of milk for de novo (C4 to C14), mixed (C16, C16:1, and C17), and preformed (≥C18) FA, as described in Woolpert et al. (2016). By dividing the grams/100 g of milk of each FA group by the sum of the 3 FA groups, in grams/100 g of milk, we calculated rel% for each FA group. As described by Wojciechowski and Barbano, (2016), we used gas-liquid chromatography reference chemistry to calibrate the milk FA parameters. The main milk constituents and FA were calibrated using the same 14-sample calibration set. Partial least squares models developed by Delta Instruments were used to predict concentrations of mpbNEFA, milk BHB, and milk acetone using parameters #1603, #1601, and #1602, respectively, while MUN was predicted using parameter #502 and calibrated using an enzymatic MUN assay (Portnoy et al., 2021) to generate reference chemistry values.

### Statistical Analysis

All descriptive statistics and statistical modeling were performed using Stata v17 (College Station, TX).

#### Calcium Dynamic Groups.

Calcium dynamic group classification was performed using logistic regressions to derive receiver operating characteristic curves. These curves were developed to obtain an optimized tCa cut point for 1 and 4 DIM separately. Cut points were tested in 0.01 mmol/L increments and were selected for use in the final models where the area under the curve (**AUC**), sensitivity and specificity were all maximized.

The 1 DIM tCa cut point was determined using long-term production data similar to Neves et al. (2018). An association was found between decreased tCa on 1 DIM and increased average daily milk yield over the first 15 weeks of lactation using linear regression with the fixed effect of parity. This association isolated a biologically relevant window of tCa from 1.93 to 2.05 mmol/L (all *P* < 0.05), where lower tCa was associated with higher milk production. Testing individual cut points within this window using logistic regression with the fixed effects of 15-wk milk yield, parity and a parity × milk yield interaction, we found that a 1.98 mmol/L tCa cut point maximized the AUC at 0.72 with both sensitivity and specificity at 69%.

The cut point at 4 DIM was determined using health data as in Neves et al. (2018). As the average DIM of movement from the early-lactation pen was 12 DIM, events of metritis, hyperketonemia, displaced abomasum, and culling (sold or died) <13 DIM were pooled as an early-lactation negative health outcome variable. Using tCa at 4 DIM to predict negative health outcomes by 12 DIM, via logistic regression, a tCa cut point of 2.22 mmol/L maximized the AUC at 0.79 with sensitivity at 76% and specificity at 80%.

Cows were subsequently classified as follows: NC = 1 DIM tCa >1.98 mmol/L and 4 DIM tCa >2.22 mmol/L; tSCH = 1 DIM tCa ≤ 1.98 mmol/L and 4 DIM tCa >2.22 mmol/L; pSCH = 1 DIM tCa ≤ 1.98 mmol/L and 4 DIM tCa ≤2.22 mmol/L; and dSCH = 1 DIM tCa >1.98 mmol/L and 4 DIM tCa ≤2.22 mmol/L.

#### Milk Yield and Constituents.

Characterization of milk yield and constituent means over time (3 to 10 DIM) among Ca dynamic groups was determined via linear regression. Models included the main effects of DIM, Ca dynamic group, and parity group (2, 3, ≥4), as well as the interactions of DIM × Ca dynamic group and parity × Ca dynamic group. Models were fitted using a backward stepwise process in which potential covariates were eliminated from the model if *P* > 0.05. However, as the variables of interest, DIM, Ca dynamic group, and their interaction term were retained in every model regardless of statistical significance. Models were adjusted using the cluster option for linear regression in Stata, with observations clustered by cow, to account for intracow correlation between repeated samples taken from the same individual (Rogers, 1993; Williams, 2000). Results are presented as marginal means with 95% confidence intervals calculated from standard errors of the predicted means.

Pairwise comparisons of Ca dynamic group means for each constituent metric, at each individual DIM, were conducted using linear regression, with the main effect of Ca dynamic group, and including parity and the interaction of parity × Ca dynamic group as potential covariates within the model. The models were fitted using the same backward stepwise process with Ca dynamic group maintained in each model as above. A Tukey correction for 4 group means was used to adjust for multiple comparisons, at each DIM.

The means of each constituent metric over the entire sample period were compared by linear regression, using the same backward stepwise process described above for comparisons at each individual DIM. Observations were again clustered by cow. For pairwise comparisons of group means over the entire sample period, a Bonferroni correction for 4 group means was used to adjust for multiple comparisons.

After modeling, the residuals were tested for normality visually using histograms and Q-Q plots as well as statistically using a Kolmogorov-Smirnov test. Constituents found to have non-normal residuals at any given day or throughout the sample period were transformed to normality using the natural logarithm function, and back transformed after modeling for presentation.

To compare the trends of daily constituent differences between Ca dynamic groups, the percentage of days at which one group was different from another with respect to any given constituent was calculated by dividing the total number of days where a difference occurred by the total number of days that the cows were sampled. The composite percentage of days where a difference occurred was calculated by taking the average of the percentages for all constituents that differed at more than one day.

## RESULTS

### Descriptive Statistics

We enrolled 399 multiparous Holsteins into our study at 1 DIM from which we collected 2,752 milk samples. Of these cows we excluded individuals with previous days carried calf <260 (n = 5), those diagnosed with a disease of interest before 4 DIM (n = 16), those missing tCa data at either 1 or 4 DIM (n = 22), those moved from the early-lactation pen before 3 DIM (n = 10), and those that did not have valid milk data for any day between 3 and 10 DIM (n = 3). We then excluded individual milk samples if they were collected from cows that had been excluded for the reasons noted above (n = 65), if they were collected before 3 DIM or after 10 DIM (n = 461), or if their FTIR data could not be validated (n = 509). After exclusion, 343 cows with 1,717 milk samples were retained for the final analysis.

Within this study population, 42.0% of cows were NC (n = 144), 36.7% were tSCH (n = 126), 9.0% were pSCH (n = 31), and 12.2% were dSCH (n = 42). Table 1 contains descriptive statistics outlining the group and population characteristics, including average daily yield per milking, average daily milk yield over 15 weeks of lactation, a breakdown of cows by parity, disease incidences within the population, and the number of cows sampled on each DIM.

### Milk Analysis

**Figure 1.**
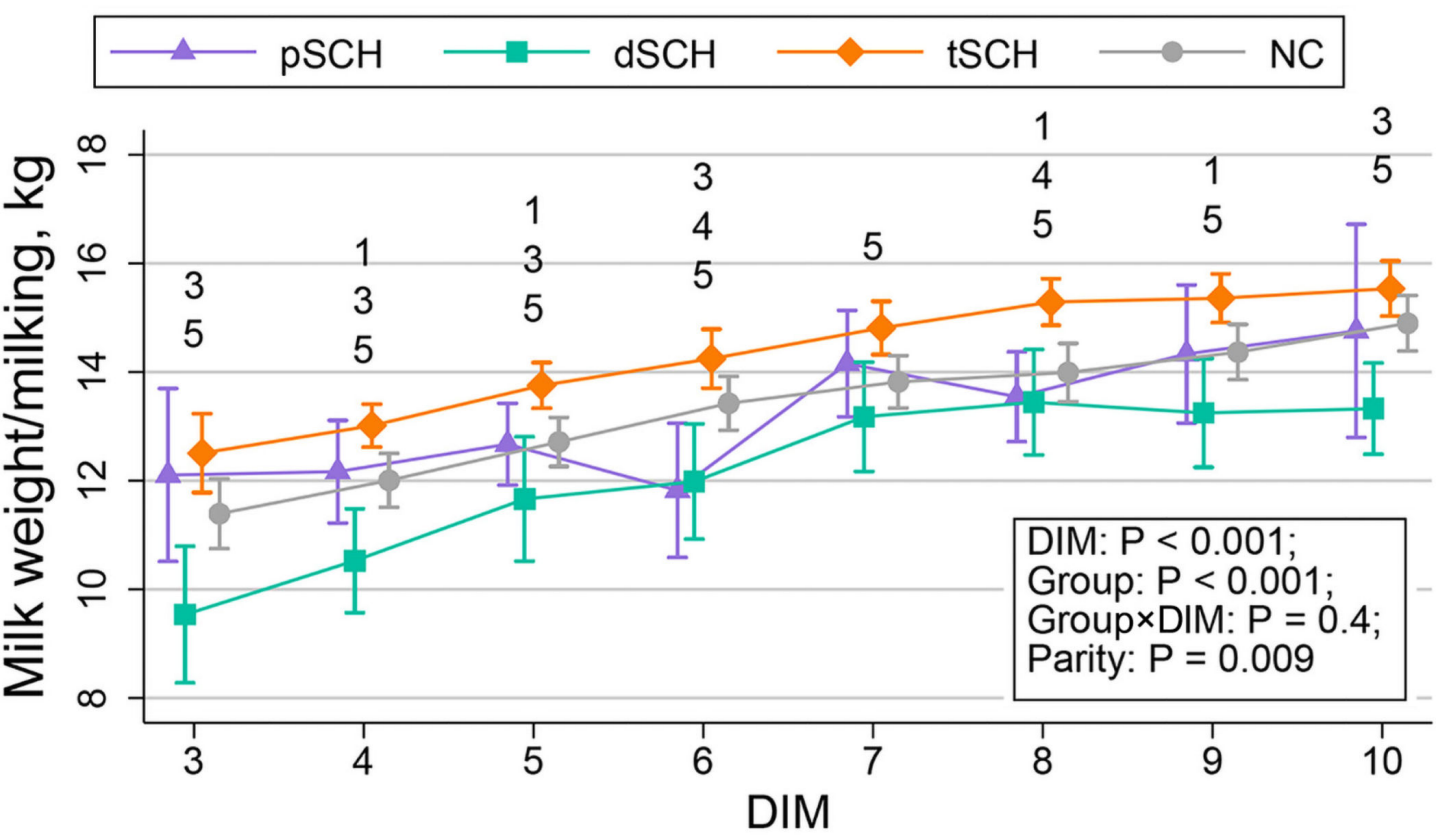
Modeled milk weight per milking from 3 through 10 DIM by Ca dynamic group, for 343 multiparous Holsteins on a commercial dairy in Cayuga County, New York. The Ca dynamic group classification was based on subclinical hypocalcemia (SCH) status at 1 DIM (total serum Ca concentration [tCa] ≤1.98 mmol/L) and 4 DIM (tCa ≤2.22 mmol/L). Groups were defined as normocalcemic (NC) or experiencing transient (tSCH; SCH at 1 DIM only), persistent (pSCH; SCH at 1 and 4 DIM), or delayed SCH (dSCH; SCH at 4 DIM only). Numbers indicate statistical differences among groups at *P* < 0.05 (corrected for multiple comparisons using Tukey’s method) at individual DIM such that 1: NC ≠ tSCH; 2: NC ≠ pSCH; 3: NC ≠ dSCH; 4: tSCH ≠ pSCH; 5: tSCH ≠ dSCH; 6: pSCH ≠ dSCH. Error bars represent 95% confidence intervals calculated from standard errors of predicted means.

**Figure 2.**
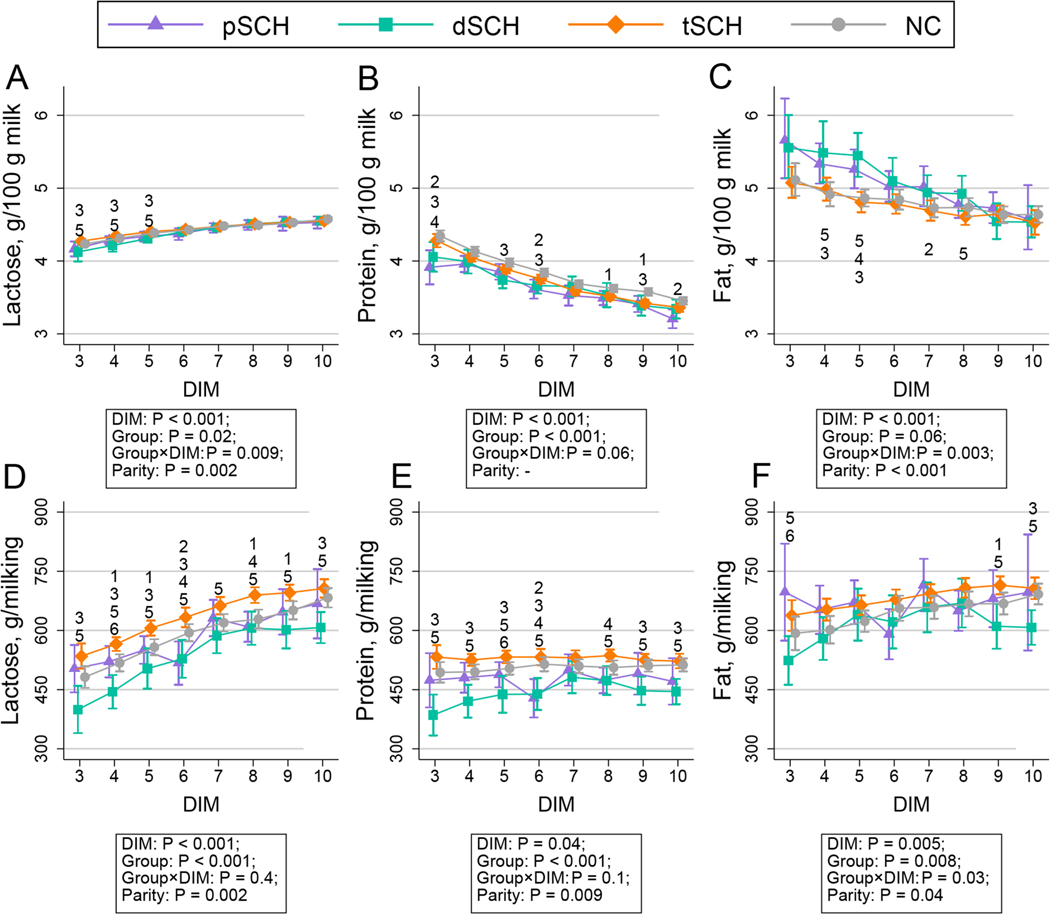
Modeled constituent means (±95% CI calculated from SE of predicted means) for lactose grams/100 g of milk and grams/milking (A and D, respectively), protein grams/100 g of milk and grams/milking (B and E, respectively), and fat grams/100 g of milk and grams/milking (C and F, respectively) predicted by Fourier-transform infrared analysis from 3 through 10 DIM by Ca dynamic group, for a study population of 343 multiparous Holsteins on a commercial dairy in Cayuga County, New York. Classification based on subclinical hypocalcemia (SCH) status at 1 DIM (total serum Ca concentration [tCa] <1.98 mmol/L) and 4 DIM (tCa <2.22 mmol/L). Groups defined as normocalcemic (NC) and cows experiencing transient (tSCH; SCH at 1 DIM only), persistent (pSCH; SCH at 1 and 4 DIM), and delayed SCH (dSCH; SCH at 4 DIM only). Numbers indicate statistical differences among groups at *P* < 0.05 (corrected for multiple comparisons using Tukey’s method) at individual DIM such that 1: NC ≠ tSCH; 2: NC ≠ pSCH; 3: NC ≠ dSCH; 4: tSCH ≠ pSCH; 5: tSCH ≠ dSCH; 6: pSCH ≠ dSCH.

**Figure 3.**
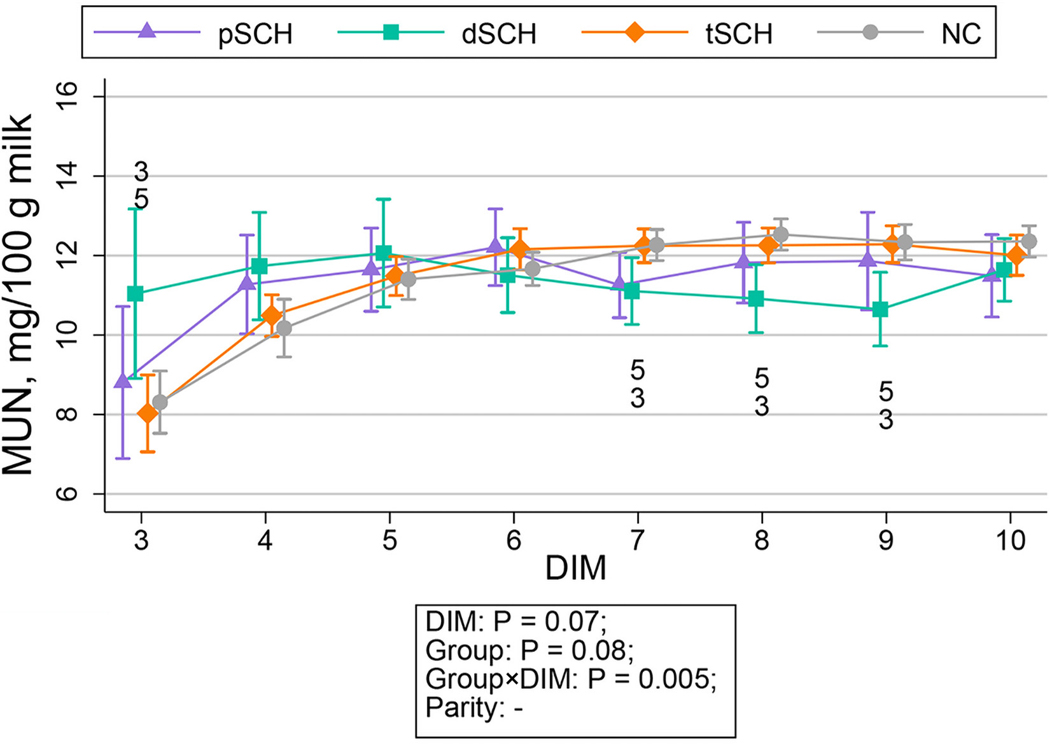
Modeled constituent means (±95% CI calculated from SE of predicted means) for MUN milligrams/100 g of milk predicted by Fourier-transform infrared analysis from 3 through 10 DIM by Ca dynamic group, for a study population of 343 multiparous Holsteins on a commercial dairy in Cayuga County, New York. Classification based on subclinical hypocalcemia (SCH) status at 1 DIM (total serum Ca concentration [tCa] <1.98 mmol/L) and 4 DIM (tCa <2.22 mmol/L). Groups defined as normocalcemic (NC) and cows experiencing transient (tSCH; SCH at 1 DIM only), persistent (pSCH; SCH at 1 and 4 DIM), and delayed SCH (dSCH; SCH at 4 DIM only). Numbers indicate statistical differences among groups at *P* < 0.05 (corrected for multiple comparisons using Tukey’s method) at individual DIM such that 1: NC ≠ tSCH; 2: NC ≠ pSCH; 3: NC ≠ dSCH; 4: tSCH ≠ pSCH; 5: tSCH ≠ dSCH; 6: pSCH ≠ dSCH.

**Figure 4.**
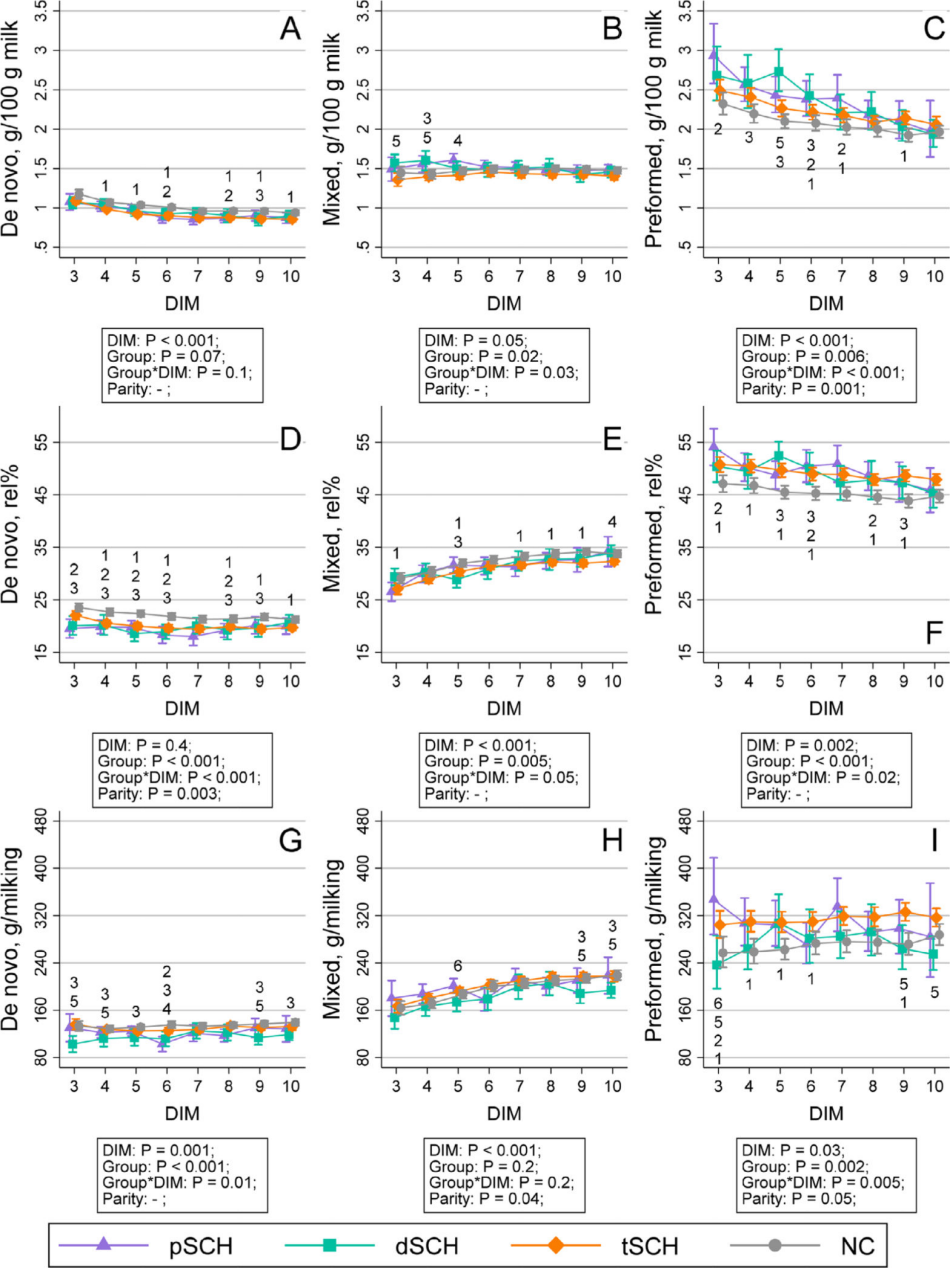
Modeled constituent means (±95% CI calculated from SE of predicted means) for de novo fatty acids (FA) grams/100 g of milk, relative percentage (rel%), and grams/milking (A, D, and G, respectively), mixed FA grams/100 g of milk, rel%, and grams/milking (B, E, and H, respectively), and preformed FA grams/100 g of milk, rel%, and grams/milking (C, F, and I, respectively) predicted by Fourier-transform infrared analysis from 3 through 10 DIM by Ca dynamic group, for a study population of 343 multiparous Holsteins on a commercial dairy in Cayuga County, New York. Classification based on subclinical hypocalcemia (SCH) status at 1 DIM (total serum Ca concentration [tCa] <1.98 mmol/L) and 4 DIM (tCa <2.22 mmol/L). Groups defined as normocalcemic (NC) and cows experiencing transient (tSCH; SCH at 1 DIM only), persistent (pSCH; SCH at 1 and 4 DIM), and delayed SCH (dSCH; SCH at 4 DIM only). Numbers indicate statistical differences among groups at *P* < 0.05 (corrected for multiple comparisons using Tukey’s method) at individual DIM such that 1: NC ≠ tSCH; 2: NC ≠ pSCH; 3: NC ≠ dSCH; 4: tSCH ≠ pSCH; 5: tSCH ≠ dSCH; 6: pSCH ≠ dSCH.

**Figure 5.**
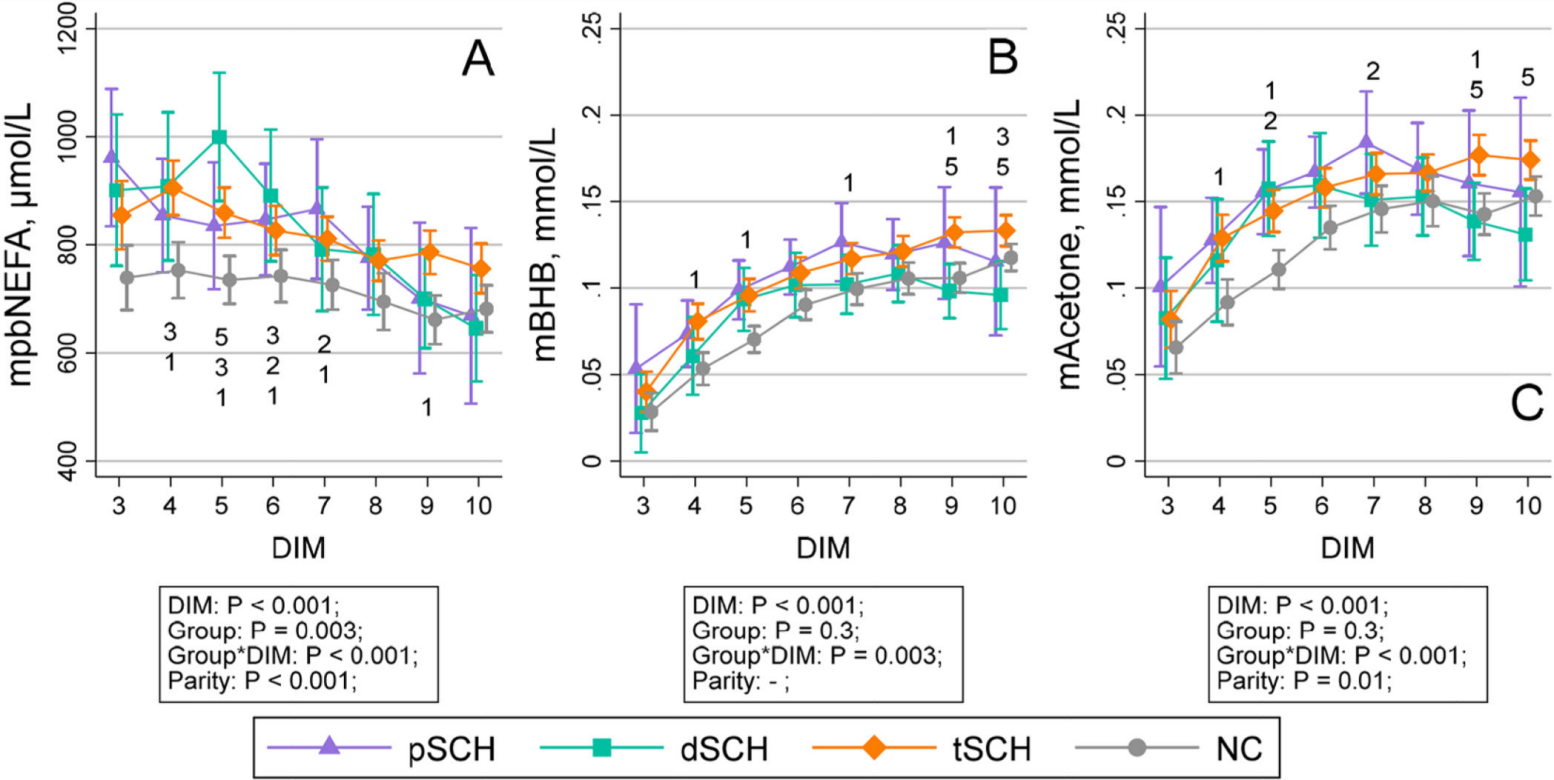
Modeled constituent means (±95% CI calculated from SE of predicted means) for (A) milk-predicted blood nonesterified fatty acids (μmol/L; mpbNEFA), (B) milk β-hydroxybutyrate (mBHB), and (C) milk acetone (mAcetone) predicted by Fourier-transform infrared analysis from 3 through 10 DIM by Ca dynamic group, for a study population of 343 multiparous Holsteins on a commercial dairy in Cayuga County, New York. Classification based on subclinical hypocalcemia (SCH) status at 1 DIM (total serum Ca concentration [tCa] <1.98 mmol/L) and 4 DIM (tCa <2.22 mmol/L). Groups defined as normocalcemic (NC) and cows experiencing transient (tSCH; SCH at 1 DIM only), persistent (pSCH; SCH at 1 and 4 DIM), and delayed SCH (dSCH; SCH at 4 DIM only). Numbers indicate statistical differences among groups at *P* < 0.05 (corrected for multiple comparisons using Tukey’s method) at individual DIM such that 1: NC ≠ tSCH; 2: NC ≠ pSCH; 3: NC ≠ dSCH; 4: tSCH ≠ pSCH; 5: tSCH ≠ dSCH; 6: pSCH ≠ dSCH.

The change in milk yield per milking over time by Ca dynamic group is displayed in Figure 1. The temporal patterns of milk constituents across Ca dynamic groups are shown in Figures 2, 3, 4, and 5. We detected an effect of DIM for all milk constituents (all *P* < 0.05) except milk urea nitrogen milligrams/100 g of milk (*P* = 0.07), mixed FA grams/100 g of milk (*P* = 0.05), and de novo FA rel% (*P* = 0.4). The parity × Ca dynamic group interaction was not statistically significant (all *P* > 0.05) for any constituent when DIM was included in the model, although this interaction did become statistically significant for some constituents when conducting pairwise comparisons at individual DIM. Modeled means and 95% confidence intervals for these pairwise comparisons examining differences among Ca dynamic groups at each DIM are presented numerically in Supplemental Table S2 (https://blogs.cornell.edu/jessmcartlab/research/supp/ca-milk/tab-2/). Modeled mean constituent concentrations over the entire sample period from 3 to 10 DIM are contained in Supplemental Table S3 (https://blogs.cornell.edu/jessmcartlab/research/supp/ca-milk/tab-3/).

#### Persistent Versus Delayed Subclinical Hypocalcemia.

The milk of pSCH cows and the milk of dSCH cows did not differ statistically at more than one time point for any constituent. These groups differed at a single time point only for yields of lactose, protein, fat, mixed FA, and preformed FA in grams/milking.

#### Normocalcemic Versus Delayed Subclinical Hypocalcemia.

The milk of NC cows differed from the milk of dSCH cows at more than one time point for all constituents other than fat yield (g/milking), de novo FA concentration by weight (g/100 g of milk), mixed FA concentration by weight (g/100 g of milk) and relative percentage, and BHB concentration by volume (mmol/L), which all differed at only one time point, as well as preformed FA yield (g/milking) and acetone concentration by volume (mmol/L) which did not differ between NC and dSCH cows’ milk at any time point. Constituents with a statistically significant difference between NC and dSCH milk at more than one time point were different for 49.0% (3.9 d) of the sample period, on average. The constituents that differed most between these 2 groups were protein yield (g/milking), and de novo FA relative percentage and yield (g/milking), which differed at 75% of time points (6 d). Additionally, lactose concentration by weight (g/100 g of milk) and yield (g/milking), protein concentration by weight (g/100 g of milk), MUN concentration by weight (mg/100 g of milk), both concentration by weight (g/100 g of milk) and relative percentage of preformed FA, and mpbNEFA (μmol/L), all showed statistically significant differences on 3 or more days. The milk yield per milking differed between NC and dSCH cows at 62.5% of milkings (5 d), with NC cows producing more than dSCH cows when we observed a difference.

#### Normocalcemic Versus Persistent Subclinical Hypocalcemia.

Milk of NC cows was not statistically different from the milk of pSCH cows for lactose concentration by weight (g/100 g of milk), fat yield (g/milking), MUN concentration by weight (mg/100 g of milk), mixed FA concentration by weight (g/100 g of milk), mixed FA relative percentage, mixed FA yield (g/milking), or BHB concentration by volume (mmol/L), and differed at only one day for lactose yield (g/milking), protein yield (g/milking), fat concentration by weight (g/100 g of milk), de novo FA yield (g/milking), and preformed FA yield (g/milking). All other constituents differed at an average of 35.7% of time points (2.9 d). Between these 2 groups, de novo FA relative percentage differed most frequently at 62.5% of time points (5 d), with NC cows having greater relative percentages of this constituent in their milk. Beyond this, NC cows had higher concentrations by weight of protein (g/100 g of milk) and lower concentrations by weight of preformed FA (g/100 g of milk) in their milk than pSCH cows on 3 or more days. These groups differed at 0% of time points with respect to milk weight.

#### Transient Versus Delayed Subclinical Hypocalcemia.

Though the milk of dSCH cows was not different from the milk of tSCH cows with respect to protein concentration by weight (g/100 g of milk), de novo FA concentration by weight (g/100 g of milk), de novo FA relative percentage, mixed FA relative percentage, or preformed FA relative percentage, and differed at only one day for preformed FA concentration by weight (g/100 g of milk), and mpbNEFA (μmol/L), the milk differed at an average of 43.8% of time points (3.5 d) for all other constituents. Between these groups, the constituents that differed most frequently were lactose yield (g/milking) and protein yield (g/milking) which differed at 100% (8 d) and 87.5% (7 d) of time points respectively, with tSCH cows yielding more of these constituents than dSCH cows. Milk urea nitrogen (mg/100 g of milk) differed at 50% of time points, where tSCH cows had higher concentrations of this constituent later in the sampling period. Lactose concentration by weight (g/100 g of milk), fat yield (g/milking), de novo FA yield (g/milking), and preformed FA yield (g/milking) all differed at 3 or more time points. The tSCH group also produced more milk than dSCH cows at 100% of milkings.

#### Transient Versus Persistent Subclinical Hypocalcemia.

Regarding lactose yield (g/milking) and protein yield (g/milking), only 25% of time points (2 d) showed a difference between the milk of pSCH and tSCH cows. The yield and concentrations of constituents in the milk of these 2 groups differed at one day or not at all for all other constituents. In addition, milk weight per milking was higher for the tSCH cows when compared with pSCH cows at only 25% of time points (2 d).

#### Normocalcemic Versus Transient Subclinical Hypocalcemia.

Lactose concentration by weight (g/100 g of milk), protein yield (g/milking), fat concentration by weight (g/100 g of milk) and yield (g/milking), MUN concentration by weight (mg/100 g of milk), de novo FA yield (g/milking) and mixed FA concentration by weight (g/100 g of milk) and yield (g/milking) differed at one time point or did not differ between the NC and tSCH groups. All other constituents differed for an average of 55.7% of the sample period (4.5 d). De novo FA concentration by weight (g/100 g of milk), de novo FA relative percentage, mixed FA relative percentage, preformed FA relative percentage, preformed FA yield (g/milking) and mpbNEFA (μmol/L) all differed for 6 or more days of the sample period, with the metrics of de novo FA and mixed FA being elevated in the milk of NC cows while preformed FA and mbpNEFA were elevated in tSCH cows. Both ketone bodies were at higher concentrations by volume (mmol/L) in tSCH milk for 3 or more days when compared with NC milk. Transiently SCH cows also produced statistically more milk and lactose (g/milking) than NC cows at 50% of milkings (4 d).

## DISCUSSION

Our objective was to characterize the milk constituent profiles of early-lactation multiparous Holsteins with differing Ca dynamics to investigate the diagnostic potential of FTIR milk analysis methods for identifying cows with unfavorable Ca dynamics in the early-postpartum period. To accomplish this objective, we needed to first classify cows into Ca dynamic groups based on population specific data on health and production outcomes.

In our study population, low tCa at 1 DIM, based on a cut point of tCa ≤1.98 mmol/L, was associated with greater average milk production over the first 15 weeks of lactation. Across study designs, variations in cut point determination strategy, and a range of sample sizes, similar trends have been observed in multiparous Holsteins experiencing SCH at a very early time point postpartum by Jawor et al. (2012; n = 69, tCa ≤1.80 mmol/L), Neves et al. (2018; n = 259, tCa ≤1.77 mmol/L), and Seely et al. (2021; n = 78, tCa ≤1.95 mmol/L). Menta et al. (2021) even found this association in herd of 380 multiparous Jersey cows. Despite discrepancies between these studies, the overall theme remains the same: for some cows, early-postpartum SCH is associated with greater long-term production. Whether this increased plane of production is the cause or result of early-postpartum SCH remains a valid question for future research.

Negative outcomes are associated with SCH when reduced tCa concentrations persist beyond the first day of lactation. Caixeta et al. (2017) found that chronic SCH, defined as tCa ≤2.15 mmol/L at all 3 of the first 3 DIM, was associated with poor reproductive performance. McArt and Neves (2020) found that SCH at 4 DIM was associated with increased risk of culling and disease. Venjakob et al. (2021) found that cows with metritis and a comorbidity had lower tCa than healthy cows at 3 DIM. Though these are not comparable studies, each assessed the dynamics of Ca throughout the early-postpartum period, rather than at a single time point, and concluded that different dynamics are likely to be differentially associated with negative outcomes. In our study, tCa ≤2.22 mmol/L at 4 DIM predicted negative health events within 12 DIM with an AUC of 0.79, which does correspond to a useful discrimination ability according to Swets (1988). We found no association between disease and tCa at 1 DIM, affirming that different Ca dynamics are differentially associated with risk of disease, such that cows experiencing normocalcemia and transient SCH are less at risk than those experiencing either persistent or delayed SCH.

Though a more minor objective of our project, providing evidence in support of the Ca dynamic group theory proposed by Neves et al. (2018) was an important byproduct of our analysis. Through this classification framework we were then able to examine our main objective.

We found that differences were present among the milk constituent profiles of cows with different Ca dynamics throughout 10 DIM, and at both 3 and 4 DIM when clinical interventions might be efficacious. We hypothesized that these profiles would be different with respect to FA groups as well as other indicators of health such as mpbNEFA and ketone bodies, but that lactose and protein would be the same across groups. Whereas the data presented here support our hypotheses regarding FA and mpbNEFA, which were different among groups at the majority of time points, our data did not support our hypotheses regarding lactose, protein and ketone bodies.

Throughout the sample period, the Ca dynamic groups had similar concentrations by weight of lactose and protein in their milk. On a yield basis, however, both the NC and tSCH groups produced more protein and lactose in their milk than the dSCH group on average and at the majority of milkings. It is known that tSCH and NC cows eat more than dSCH and pSCH cows (Seely et al., 2021) thus it is likely that these cows have a greater supply of gluconeogenic precursors, leading to increased capacity for lactose production in the mammary gland through increased blood glucose concentrations. It is also known that the mammary gland has an exceptionally large capacity to efficiently uptake Ca from the blood (Montalbetti et al., 2014), and the fusion of lactose- and protein-containing intracellular vesicles with the apical membrane of mammary epithelial cells is Ca dependent (Truchet et al. 2014). Therefore, it is possible that increased tCa drives an influx of Ca into the cell that might be responsible for increased vesicle fusion and thereby increased secretion of these components into milk. Further research directed at elucidating these mechanisms is certainly warranted.

When we examined the metrics of FA groups, we found that throughout the sampling period we observed differences among Ca dynamic groups with respect to FA concentrations by weight, rel%, and yield. Notably, the milk of NC cows had higher rel% of de novo FA when compared with the milk of tSCH, pSCH, and dSCH cows, at the majority of time points. Normocalcemic cows also had greater de novo FA yields per milking than dSCH cows at the majority of time points but yield of de novo FA was not different between NC and tSCH cows at any time point. In previous research, it has been shown that the milk of cows in excessive energy deficit, and that of cows with hyperketonemia, have a FA profile characterized by low de novo FA and high preformed FA (Gross et al., 2011; Seely et al., 2022). Cows at greater risk of disease or culling have also been shown to have lower levels of de novo FA in their milk (Bach et al., 2019). These findings indicate that de novo FA may be associated with health status. Barbano et al. (2014), found a strong correlation between bulk tank de novo FA concentrations and bulk tank true protein, and proposed the hypothesis that de novo FA may be linked to a healthy rumen microbial environment. Our observations that NC cows in this study population were at decreased risk of negative health events and had consistently greater levels of de novo FA when compared with dSCH cows fits well with this hypothesis. Combined with the knowledge that NC and tSCH cows eat more than the other groups (Seely et al., 2021), it seems likely that conditions in the rumen are mechanistically related to these observations, although further evidence would be needed to support that theory.

We would have expected based on the findings of Chamberlin et al. (2013) and Bach et al. (2019) that cows with unfavorable Ca dynamics, dSCH and pSCH cows, would have had higher concentrations of mpbNEFA than NC cows. We did not see this trend, but it is possible that was due to the relatively small proportion of animals in these at-risk groups in our study or differences in the design and the populations sampled in those studies. Preformed FA rel% and yield, as well as mpbNEFA were both consistently elevated for the milk of tSCH cows when compared with NC cows. In the literature we detected strong evidence that high blood NEFA concentrations are associated with negative outcomes (Herdt, 2000; Contreras and Sordillo, 2011; Tremblay et al., 2018), so it is curious that our most productive and healthy group had the highest predicted value for this constituent. Though the mechanism behind these observations is beyond the scope of this study, these findings may simply be reflective of the increased energy demands placed on tSCH cows to support the copious milk production we observed. Based on the understanding that preformed FA in milk originate from the diet or from mobilized fat reserves (Palmquist, 2006; Stoop et al., 2009), it is possible that enough NEFA from the blood were imported into mammary cells to mitigate the negative outcomes associated with high blood NEFA, by preventing NEFA from progressively accumulating in the blood. It is also plausible that the liver function of tSCH cows is improved compared with the liver function of the other groups resulting in greater capacity for complete oxidation of NEFA from the blood and more efficient utilization of these compounds for energy (White, 2015). The evidence of increased gluconeogenesis in the livers of tSCH cows, indicated by increased yields of lactose in their milk, also supports this hypothesis. This mechanism might explain both decreased negative health outcomes and higher milk production observed in tSCH cows, but further research is needed to understand the mechanisms by which these tSCH cows cope with increased metabolic stress so adeptly.

Ketone bodies were not different among groups. Though numerical differences existed on certain days, there was not a strong statistical association between ketones in milk and Ca status. Furthermore, where we observed a statistical difference in ketone concentrations, the biological relevance of this difference is questionable. As with mpbNEFA, we would have expected our at-risk groups to have had higher levels of these metabolites in their milk based on previous research (Bach et al., 2019). The finding that they did not may be a signal that ketones are not a good indicator of Ca status, despite their connection to negative health events. It is also possible that our study was underpowered to evaluate differences in ketone bodies.

Our study is limited by sampling on a single farm, with a single diet, in a single season, and therefore these patterns that we have described may simply be the product of a unique population, management scheme or seasonal effect. We did not track the intake of these cows and it is difficult to determine whether the differences observed may link back to changes in intake as shown in Seely et al. (2021). In addition, samples at 3 DIM were disproportionately excluded on the basis of their invalid FTIR data, likely due to the unique composition of transition milk. However, despite our limitations, these findings together support the use of FTIR as a potential diagnostic method. If future work can further characterize these patterns in other populations, it may be possible to develop a method for identifying cows of favorable or unfavorable Ca dynamics that could be used to influence management and clinical interventions.

## CONCLUSIONS

Constituent profiles for cows of differing Ca dynamics were distinct throughout the sampling period. Despite sampling on a single farm, our findings indicate that FTIR methods show promise as a method for discriminating between healthy and less healthy cows as early as 3 or 4 DIM, and for capturing the variability in cows with respect to Ca dynamics. Future work should focus on further characterizing these patterns on multiple farms and investigating the potential implications for management changes.

## Supplementary Material

Supplemental Table 1

Supplemental Table 2

Supplemental Table 3

## Figures and Tables

**Table 1.** Descriptive characteristics of the study population of multiparous Holstein cows (n = 343) on a freestall dairy in Cayuga County, New York, classified into Ca dynamic groups based on subclinical hypocalcemia (SCH) status at 1 DIM (tCa ≤1.98 mmol/L) and 4 DIM (tCa ≤2.22 mmol/L)^1^

Item	Ca dynamic group				Total
	NC	tSCH	pSCH	dSCH	

Cows, n	144	126	31	42	343
Milk yield/milking,^2^ kg	13.5 ± 2.6	14.3 ± 2.4	13.0 ± 2.7	12.3 ± 3.0	13.6 ± 2.7
15-wk average milk yield/d,^3^ kg	51.9 ± 7.5	54.5 ± 7.4	50.9 ± 11.9	50.2 ± 8.4	52.6 ± 8.2
Parity,^4^ n (%)					
2	77 (53)	39 (31)	5 (16)	20 (48)	141 (41)
3	47 (33)	34 (27)	12 (39)	15 (36)	108 (31)
≥4	20 (14)	53 (42)	14 (45)	7 (17)	94 (27)
Disease incidence,^5^ n (%)					
Metritis	4 (3)	1 (1)	3 (10)	9 (21)	17 (5)
Hyperketonemia	7 (5)	14 (11)	14 (32)	9 (21)	40 (12)
Displaced abomasum	1 (1)	2 (2)	2 (6)	1 (2)	6 (2)
Culled	11 (8)	13 (10)	7 (23)	6 (14)	37 (11)
Early-lactation disease^6^	8 (6)	10 (8)	12 (39)	19 (45)	49 (14)
Milk samples according to DIM,^7^ n (%)					
3	65 (41)	59 (38)	12 (8)	21 (13)	157
4	88 (41)	83 (39)	19 (9)	24 (11)	214
5	107 (45)	85 (35)	21 (9)	27 (11)	240
6	105 (43)	87 (36)	23 (10)	27 (11)	242
7	89 (41)	85 (39)	20 (9)	25 (11)	219
8	90 (41)	83 (38)	22 (10)	24 (11)	219
9	93 (43)	85 (39)	12 (6)	27 (12)	217
10	96 (46)	76 (36)	11 (5)	26 (12)	209
Total milk samples	733 (43)	643 (37)	140 (8)	201 (12)	1,717

1 Groups defined cows as normocalcemic (NC) or experiencing transient (tSCH; SCH at 1 DIM only), persistent (pSCH; SCH at 1 and 4 DIM), or delayed SCH (dSCH; SCH at 4 DIM only). tCa = total serum Ca concentration.

2 Mean of all recorded milk weights from 3 to 10 DIM by Ca dynamic group ± SD.

3 Mean of daily milk weight averages by week for 15 wk of lactation for cows retained in the herd ± SD.

4 Total cows in each parity group by Ca dynamic group (percentage of Ca dynamic group in each parity).

5 Total cows diagnosed with disease of interest (incidence of disease of interest by Ca dynamic group).

6 Defined as farm-diagnosed metritis, hyperketonemia (blood BHB ≥ 1.2 mmol/L), displaced abomasum, or culling (sold or died) between 3 to 12 DIM.

7 Total milk samples collected at each DIM by Ca dynamic group (percentage of total milk samples at each DIM).

## References

[R1] Bach KD, Barbano DM, and McArt JAA 2019. Association of mid-infrared-predicted milk and blood constituents with early-lactation disease, removal, and production outcomes in Holstein cows. J. Dairy Sci. 102:10129–10139. 10.3168/jds.2019-16926. 31495624

[R2] Barbano DM, Melilli C, and Overton TR 2014. Advanced use of FTIR spectra of milk for feeding and health management. Pages 105–113 in Proc. Cornell Nutrition Conf., Syracuse, NY. Cornell Univ., Dept. of Animal Sci., Ithaca, NY.

[R3] Bauman DE, and Currie WB 1980. Partitioning of nutrients during pregnancy and lactation: A review of mechanisms involving homeostasis and homeorhesis. J. Dairy Sci. 63:1514–1529. 10.3168/jds.S0022-0302(80)83111-0. 7000867

[R4] Caixeta LS, Ospina PA, Capel MB, and Nydam DV 2017. Association between subclinical hypocalcemia in the first 3 days of lactation and reproductive performance of dairy cows. Theriogenology 94:1–7. 10.1016/j.theriogenology.2017.01.039. 28407850

[R5] Callero KR, Teplitz EM, Barbno DM, Seely CR, Seminara JA, Frost IR, McCray HA, Martinez RM, Reid AM, and McArt JAA 2023. Patterns of Fourier-transform infrared estimated milk constituents in early lactation Holstein cows. J. Dairy Sci. 106:2716–2728. 10.3168/jds.2022-22588. 36823015 PMC10957286

[R6] Chamberlin WG, Middleton JR, Spain JN, Johnson GC, Ellersieck MR, and Pithua P 2013. Subclinical hypocalcemia, plasma biochemical parameters, lipid metabolism, postpartum disease, and fertility in postparturient dairy cows. J. Dairy Sci. 96:7001–7013. 10.3168/jds.2013-6901. 24054301

[R7] Chapinal N, LeBlanc SJ, Carson ME, Leslie KE, Godden S, Capel M, Santos JEP, Overton MW, and Duffield TF 2012. Herd-level association of serum metabolites in the transition period with disease, milk production, and early lactation reproductive performance. J. Dairy Sci. 95:5676–5682. 10.3168/jds.2011-5132. 22863094

[R8] Contreras GA, and Sordillo LM 2011. Lipid mobilization and inflammatory responses during the transition period of dairy cows. Comp. Immunol. Microbiol. Infect. Dis. 34:281–289. 10.1016/j.cimid.2011.01.004. 21316109

[R9] Couto Serrenho R, DeVries TJ, Duffield TF, and LeBlanc SJ 2021. Graduate Student Literature Review: What do we know about the effects of clinical and subclinical hypocalcemia on health and performance of dairy cows? J. Dairy Sci. 104:6304–6326. 10.3168/jds.2020-19371. 33685698

[R10] Goff JP 2008. The monitoring, prevention, and treatment of milk fever and subclinical hypocalcemia in dairy cows. Vet. J. 176:50–57. 10.1016/j.tvjl.2007.12.020. 18342555

[R11] Goff JP, Kimura K, and Horst RL 2002. Effect of mastectomy on milk fever, energy, and vitamins A, E, and β-carotene status at parturition. J. Dairy Sci. 85:1427–1436. 10.3168/jds.S0022-0302(02)74210-0. 12146473

[R12] Gross J, van Dorland HA, Bruckmaier RM, and Schwarz FJ 2011. Milk fatty acid profile related to energy balance in dairy cows. J. Dairy Res. 78:479–488. 10.1017/S0022029911000550. 21843394

[R13] Herdt TH 2000. Ruminant adaptation to negative energy balance: Influences on the etiology of hyperketonemia and fatty liver. Vet. Clin. North Am. Food Anim. Pract. 16:215–230. 10.1016/S0749-0720(15)30102-X. 11022337

[R14] Ho PN, Luke TDW, and Pryce JE 2021. Validation of milk mid-infrared spectroscopy for predicting the metabolic status of lactating dairy cows in Australia. J. Dairy Sci. 104:4467–4477. 10.3168/jds.2020-19603. 33551158

[R15] Horst RL, Goff JP, and Reinhardt TA 1997. Calcium and vitamin D metabolism during lactation. J. Mammary Gland Biol. Neoplasia 2:253–263. 10.1023/A:1026384421273. 10882309

[R16] Jawor PE, Huzzey JM, LeBlanc SJ, and von Key-serlingk MAG 2012. Associations of subclinical hypocalcemia at calving with milk yield, and feeding, drinking, and standing behaviors around parturition in Holstein cows. J. Dairy Sci. 95:1240–1248. 10.3168/jds.2011-4586. 22365207

[R17] Jorjong S, van Knegsel ATM, Verwaeren J, Bruckmaier RM, de Baets B, Kemp B, and Fievez V 2015. Milk fatty acids as possible biomarkers to diagnose hyperketonemia in early lactation. J. Dairy Sci. 98:5211–5221. 10.3168/jds.2014-8728. 26094221

[R18] Kaylegian KE, Lynch JM, Fleming JR, and Barbano DM 2009. Influence of fatty acid chain length and unsaturation on mid-infrared milk analysis. J. Dairy Sci. 92:2485–2501. 10.3168/jds.2008-1910. 19447980

[R19] Luke TDW, Rochfort S, Wales WJ, Bonfatti V, Marett L, and Pryce JE 2019. Metabolic profiling of early-lactation dairy cows using milk mid-infrared spectra. J. Dairy Sci. 102:1747–1760. 10.3168/jds.2018-15103. 30594377

[R20] Martinez N, Risco CA, Lima FS, Bisinotto RS, Greco LF, Ribeiro ES, Maunsell F, Galvão K, and Santos JEP 2012. Evaluation of peripartal calcium status, energetic profile, and neutrophil function in dairy cows at low or high risk of developing uterine disease. J. Dairy Sci. 95:7158–7172. 10.3168/jds.2012-5812. 23021755

[R21] McArt JAA, and Neves RC 2020. Association of transient, persistent, or delayed subclinical hypocalcemia with early lactation disease, removal, and milk yield in Holstein cows. J. Dairy Sci. 103:690–701. 10.3168/jds.2019-17191. 31704009

[R22] Menta PR, Fernandes L, Poit D, Celestino ML, Machado VS, Ballou MA, and Neves RC 2021. Association of blood calcium concentration in the first 3 days after parturition and energy balance metabolites at day 3 in milk with disease and production outcomes in multiparous Jersey cows. J. Dairy Sci. 104:5854–5866. 10.3168/jds.2020-19189. 33612230

[R23] Montalbetti N, Dalghi MG, Albrecht C, and Hediger MA 2014. Nutrient transport in the mammary gland: Calcium, trace minerals, and water soluble vitamins. J. Mammary Gland Biol. Neoplasia 19:73–90. 10.1007/s10911-014-9317-9. 24567109

[R24] Neves RC, Leno BM, Bach KD, and McArt JAA 2018. Epidemiology of subclinical hypocalcemia in early-lactation Holstein dairy cows: The temporal associations of plasma calcium concentration in the first 4 days in milk with disease and milk production. J. Dairy Sci. 101:9321–9331. 10.3168/jds.2018-14587. 30077442

[R25] Palmquist DL 2006. Milk fat: Origin of fatty acids and influence of nutritional factors thereon. Pages 43–92 in Advanced Dairy Chem. Vol. 2. Lipids P. F. Fox and McSweeney PLH, ed. Springer, New York, NY. 10.1007/0-387-28813-9_2

[R26] Portnoy M, Coon C, and Barbano D 2021. Performance evaluation of an enzymatic spectrophotometric method for milk urea nitrogen. J. Dairy Sci. 104:11422–11431. 10.3168/jds.2021-20308. 34389147

[R27] Ramberg CF Jr., Johnson EK, Fargo RD, and Kronfeld DS 1984. Calcium homeostasis in cows, with special reference to parturient hypocalcemia. Am. J. Physiol. 246:R698–R704. 10.1152/ajpregu.1984.246.5.R698. 6720993

[R28] Reinhardt TA, Lippolis JD, McCluskey BJ, Goff JP, and Horst RL 2011. Prevalence of subclinical hypocalcemia in dairy herds. Vet. J. 188:122–124. 10.1016/j.tvjl.2010.03.025. 20434377

[R29] Rogers WH 1993. Regression standard errors in clustered samples. Stata Tech. Bull. 13:19–23.

[R30] Sargeant JM, O’Connor AM, Dohoo IR, Erb HN, Cevallos M, Egger M, Ersbøll AK, Martin SW, Nielsen LR, Pearl DL, Pfeiffer DU, Sanchez J,, Torrence ME, Vigre H, Waldner C, and Ward MP 2016. Methods and processes of developing the strengthening the reporting of observational studies in epidemiology−veterinary (STROBE-Vet) statement. Preventive Vet. Med. 134:188–196. 10.1016/j.prevetmed.2016.09.005. 27836042

[R31] Seely CR, Bach KD, Barbano DM, and McArt JAA 2022. Diurnal variation of milk fatty acids in early-lactation Holstein cows with and without hyperketonemia. Animal 16:100552. 10.1016/j.animal.2022.100552. 35687942

[R32] Seely CR, Leno BM, Kerwin AL, Overton TR, and McArt JAA 2021. Association of subclinical hypocalcemia dynamics with dry matter intake, milk yield, and blood minerals during the periparturient period. J. Dairy Sci. 104:4692–4702. 10.3168/jds.2020-19344. 33589249

[R33] Stoop WM, Bovenhuis H, Heck JML, and van Arendonk JAM 2009. Effect of lactation stage and energy status on milk fat composition of Holstein-Friesian cows. J. Dairy Sci. 92:1469–1478. 10.3168/jds.2008-1468. 19307628

[R34] Swets JA 1988. Measuring the accuracy of diagnostic systems. Science 240:1285–1293. 10.1126/science.3287615. 3287615

[R35] Tremblay M, Kammer M, Lange H, Plattner S, Baumgartner C, Stegeman JA, Duda J, Mansfeld R, and Döpfer D 2018. Identifying poor metabolic adaptation during early lactation in dairy cows using cluster analysis. J. Dairy Sci. 101:7311–7321. 10.3168/jds.2017-13582. 29729924

[R36] Truchet S, Chat S, and Ollivier-Bousquet M 2014. Milk secretion: The role of SNARE proteins. J. Mammary Gland Biol. Neoplasia 19:119–130. 10.1007/s10911-013-9311-7. 24264376

[R37] Venjakob PL, Staufenbiel R, Heuwieser W, and Borchardt S 2021. Association between serum calcium dynamics around parturition and common postpartum diseases in dairy cows. J. Dairy Sci. 104:2243–2253. 10.3168/jds.2019-17821. 33246622

[R38] White HM 2015. The role of TCA cycle anaplerosis in ketosis and fatty liver in periparturient dairy cows. Animals (Basel) 5:793–802. 10.3390/ani5030384. 26479386 PMC4598706

[R39] Williams RL 2000. A note on robust variance estimation for cluster-correlated data. Biometrics 56:645–646. 10.1111/j.0006-341X.2000. 10877330

[R40] Wojciechowski KL, and Barbano DM 2016. Prediction of fatty acid chain length and unsaturation of milk fat by mid-infrared milk analysis. J. Dairy Sci. 99:8561–8570. 10.3168/jds.2016-11248. 27592430

[R41] Wojciechowski KL, Melilli C, and Barbano DM 2016. A proficiency test system to improve performance of milk analysis methods and produce reference values for component calibration samples for infrared milk analysis. J. Dairy Sci. 99:6808–6827. 10.3168/jds.2016-10936. 27209129

[R42] Woolpert ME, Dann HM, Cotanch KW, Melilli C, Chase LE, Grant RJ, and Barbano DM 2016. Management, nutrition, and lactation performance are related to bulk tank milk de novo fatty acid concentration on northeastern US dairy farms. J. Dairy Sci. 99:8486–8497. 10.3168/jds.2016-10998. 27522424

