# Supplementary material for: Calcium dynamics and associated temporal patterns of milk constituents in early-lactation multiparous Holsteins
Source: J Dairy Sci. Author manuscript; Available in PMC 2025 Dec 15. (PMC12703615; doi:10.3168/jds.2022-23142)
Supplement: Supplemental Table 1 [file NIHMS2111481-supplement-Supplemental_Table_1.pdf]

Supplemental Table 1. Measured composition of ingredients and nutrients in the early lactation diet of a study population of 343 multiparous Holsteins on a commercial dairy in Cayuga County, NY. Feed was sampled at 5 different timepoints from June through July 2021. Presented as means with ± standard deviation.

	% DM[1]
Ingredients:	
Wheat straw	
Corn silage	40.0 ± 0.3
Haylage	18.8 ± 0.7
Whey blend	2.7 ± 0.0
Concentrates	36.4 ± 0.6
Nutrient composition:	
DM	45.0 ± 0.3
NE_L, Mcal/kg	1.7 ± 0.0
CP	17.7 ± 0.2
aNDF[2]	30.6 ± 0.3
Starch	23.8 ± 0.1
Ether extract	4.4 ± 0.1
Ca	0.9 ± 0.0
P	0.4 ± 0.0
Mg	0.3 ± 0.0
K	1.5 ± 0.0
S	0.3 ± 0.0
Na	0.4 ± 0.0
Cl	0.6 ± 0.0

[1]unless otherwise stated
[2]aNDF = amylase-corrected neutral detergent fiber
