# Supplementary material for: Calcium dynamics and associated temporal patterns of milk constituents in early-lactation multiparous Holsteins
Source: J Dairy Sci. Author manuscript; Available in PMC 2025 Dec 15. (PMC12703615; doi:10.3168/jds.2022-23142)
Supplement: Supplemental Table 2 [file NIHMS2111481-supplement-Supplemental_Table_2.pdf]

Supplemental Table 2. Means and 95% confidence intervals of modeled milk estimates of 343 multiparous Holsteins on a commercial dairy in Cayuga County, NY, sampled from 3 through 10 DIM by Ca dynamic group. Calcium dynamic group classification was based on subclinical hypocalcemia (**SCH**) status at 1 DIM (tCa < 1.98 mmol/L) and 4 DIM (tCa < 2.22 mmol/L). Groups defined as normocalcemic (**NC**) and cows experiencing transient (**tSCH**; SCH at 1 DIM only), persistent (**pSCH**; SCH at 1 and 4 DIM), and delayed SCH (**dSCH**; SCH at 4 DIM only).

	Calcium dynamic group				*P*-values		
	NC	tSCH	pSCH	dSCH	Group	Parity	G×P
**3 DIM**							
Cows	n= 65	n= 59	n= 12	n= 21			
Milk weight/milking, kg	11.5^{A} (10.8, 12.2)	12.5^{A} (11.8, 13.2)	11.9^{AB} (10.4, 13.5)	9.6^{B} (8.4, 10.8)	<0.001	-	-
Constituents:							
Lactose, g/100 g milk	4.24^{A} (4.20, 4.28)	4.27^{A} (4.23, 4.31)	4.15^{AB} (4.06, 4.24)	4.13^{B} (4.06, 4.20)	0.002	-	-
Lactose, g/milking	488^{A} (459, 517)	533^{A} (503, 564)	493^{AB} (425, 560)	403^{B} (352, 455)	<0.001	-	-
Protein, g/100 g milk	4.37^{A} (4.28, 4.46)	4.28^{AB} (4.19, 4.37)	3.87^{C} (3.67, 4.08)	4.07^{BC} (3.92, 4.23)	<0.001	0.03	-
Protein, g/milking	497^{A} (469, 524)	531^{A} (503, 560)	467^{AB} (403, 531)	388^{B} (339, 436)	<0.001	-	-
Fat, g/100 g milk	5.22 (4.99, 5.45)	5.15 (4.92, 5.39)	5.73 (5.20, 6.25)	5.66 (5.27, 6.06)	0.05	0.02	-
Fat, g/milking	591^{AB} (551, 631)	637^{A} (594, 679)	697^{A} (603, 790)	523^{B} (453, 594)	0.01	-	-
MUN[1], mg/100 g milk	8.31^{B} (7.40, 9.22)	8.03^{B} (7.07, 8.99)	8.80^{AB} (6.68, 10.93)	11.04^{A} (9.43, 12.65)	0.02	-	-
Fatty acids:							
De novo, g/100 g milk	1.17 (1.12, 1.22)	1.09 (1.04, 1.14)	1.08 (0.96, 1.19)	1.07 (0.98, 1.15)	0.05	-	-
De novo, rel%[2]	23.42^{A} (22.60, 24.23)	22.08^{AB} (21.24, 22.91)	19.73^{B} (17.86, 21.59)	20.01^{B} (18.61, 21.41)	<0.001	0.002	-
De novo, g/milking	133^{A} (125, 142)	136^{A} (127, 145)	130^{AB} (110, 150)	103^{B} (88, 118)	0.002	-	-
Mixed, g/100 g milk	1.45^{AB} (1.37, 1.52)	1.36^{B} (1.28, 1.44)	1.49^{AB} (1.32, 1.67)	1.57^{A} (1.44, 1.70)	0.047	-	-
Mixed, rel%	29.15^{A} (28.16, 30.13)	27.13^{B} (26.10, 28.16)	26.55^{AB} (24.26, 28.84)	29.39^{AB} (27.65, 31.12)	0.01	-	-
Mixed, g/milking	164 (153, 175)	166 (155, 178)	179 (153, 205)	148 (128, 167)	0.3	-	-
Preformed, g/100 g milk	2.33^{B} (2.20, 2.47)	2.49^{AB} (2.35, 2.65)	2.93^{A} (2.55, 3.35)	2.69^{AB} (2.43, 2.98)	0.008	0.02	-
Preformed, rel%	47.11^{B} (45.58, 48.65)	50.76^{A} (49.15, 52.37)	54.08^{A} (50.51, 57.65)	50.41^{AB} (47.71, 53.11)	<0.001	-	-
Preformed, g/milking	256^{B} (234, 279)	303^{A} (276, 333)	347^{A} (282, 427)	236^{B} (202, 276)	0.002	-	-
Energy related metabolites:							
mpbNEFA[3], µmol/L	751 (687, 815)	855 (790, 920)	947 (801, 1093)	908 (799, 1018)	0.01	<0.001	-
mBHB[4], mmol/L	0.03 (0.02, 0.04)	0.04 (0.03, 0.05)	0.05 (0.03, 0.08)	0.03 (0.01, 0.05)	0.2	-	-
mAcetone, mmol/L	0.07 (0.05, 0.09)	0.08 (0.06, 0.10)	0.10 (0.06, 0.14)	0.08 (0.06, 0.11)	0.4	0.01	-
**4 DIM**							
Cows	n= 88	n= 83	n= 19	n= 24			
Milk weight/milking, kg	12.0^{B} (11.5, 12.4)	13.0^{A} (12.6, 13.5)	12.2^{ABC} (11.2, 13.1)	10.5^{C} (9.7, 11.4)	<0.001	0.02	-
Constituents:							
Lactose, g/100 g milk	4.32^{A} (4.30, 4.35)	4.34^{A} (4.31, 4.37)	4.28^{AB} (4.22, 4.34)	4.22^{B} (4.17, 4.27)	<0.001	-	-
Lactose, g/milking	517^{B} (497, 537)	565^{A} (545, 585)	522^{AB} (480, 563)	444^{C} (407, 481)	<0.001	0.01	-

Protein, g/100 g milk	4.13 (4.07, 4.20)	4.05 (3.99, 4.12)	3.96 (3.82, 4.10)	3.99 (3.87, 4.12)	0.05	-	-
Protein, g/milking	495^{A} (477, 513)	525^{A} (508, 543)	480^{AB} (443, 518)	421^{B} (387, 454)	<0.001	0.02	-
Fat, g/100 g milk	4.99^{B} (4.82, 5.16)	5.04^{B} (4.87, 5.21)	5.36^{AB} (5.00, 5.71)	5.59^{A} (5.27, 5.90)	0.005	0.01	-
Fat, g/milking	599 (569, 629)	652 (621, 683)	654 (590, 719)	579 (521, 636)	0.03	-	-
MUN, mg/100 g milk	10.40 (9.75, 11.05)	10.51 (9.86, 11.15)	11.13 (9.78, 12.48)	11.82 (10.62, 13.02)	0.2	0.01	-
Fatty acids:							
De novo, g/100 g milk	1.07^{A} (1.04, 1.10)	0.98^{B} (0.95, 1.02)	1.02^{AB} (0.95, 1.09)	1.05^{AB} (0.99, 1.11)	0.003	-	-
De novo, rel%	22.57^{A} (21.87, 23.28)	20.56^{B} (19.86, 21.26)	19.94^{B} (18.47, 21.40)	20.19^{B} (18.89, 21.50)	<0.001	0.003	-
De novo, g/milking	127^{A} (121, 133)	128^{A} (122, 133)	124^{AB} (112, 135)	111^{B} (101, 121)	0.035	0.02	-
Mixed, g/100 g milk	1.45^{B} (1.39, 1.51)	1.40^{B} (1.34, 1.46)	1.55^{AB} (1.43, 1.66)	1.61^{A} (1.51, 1.72)	0.003	0.01	-
Mixed, rel%	30.36 (29.51, 31.21)	28.95 (28.07, 29.82)	29.97 (28.14, 31.80)	30.23 (28.60, 31.86)	0.1	-	-
Mixed, g/milking	173 (165, 181)	181 (173, 189)	187 (170, 204)	167 (151, 182)	0.2	0.02	-
Preformed, g/100 g milk	2.27^{B} (2.14, 2.41)	2.47^{AB} (2.34, 2.60)	2.60^{AB} (2.32, 2.88)	2.72^{A} (2.47, 2.97)	0.006	0.02	-
Preformed, rel%	46.74^{B} (45.39, 48.10)	50.49^{A} (49.10, 51.88)	50.29^{AB} (47.38, 53.20)	49.44^{AB} (46.85, 52.03)	0.001	-	-
Preformed, g/milking	257^{B} (240, 275)	309^{A} (288, 331)	309^{AB} (267, 357)	264^{AB} (231, 300)	0.001	-	-
Energy related metabolites:							
mpbNEFA, µmol/L	761^{B} (707, 815)	906^{A} (852, 959)	849^{AB} (737, 962)	911^{A} (811, 1011)	0.001	<0.001	-
mBHB, mmol/L	0.05^{B} (0.04, 0.06)	0.08^{A} (0.07, 0.09)	0.07^{AB} (0.05, 0.09)	0.06^{AB} (0.04, 0.08)	0.002	-	-
mAcetone, mmol/L	0.09^{B} (0.08, 0.11)	0.13^{A} (0.12, 0.14)	0.13^{AB} (0.10, 0.16)	0.12^{AB} (0.09, 0.14)	0.005	0.006	-
**5 DIM**							
Cows	n= 107	n= 85	n= 21	n= 27			
Milk weight/milking, kg	12.8^{B} (12.4, 13.3)	13.7^{A} (13.3, 14.2)	12.7^{ABC} (11.7, 13.7)	11.2^{C} (10.3, 12.1)	0.03	0.2	0.03
Constituents:							
Lactose, g/100 g milk	4.40^{A} (4.37, 4.42)	4.40^{A} (4.37, 4.43)	4.33^{AB} (4.28, 4.39)	4.32^{B} (4.27, 4.36)	0.005	-	-
Lactose, g/milking	565^{B} (544, 586)	605^{A} (584, 627)	554^{ABC} (509, 598)	484^{C} (445, 524)	0.02	0.1	0.02
Protein, g/100 g milk	3.98^{A} (3.92, 4.03)	3.89^{AB} (3.83, 3.95)	3.85^{AB} (3.73, 3.97)	3.74^{B} (3.63, 3.85)	<0.001	-	-
Protein, g/milking	505^{A} (488, 523)	531^{A} (513, 549)	491^{A} (454, 528)	419^{B} (386, 452)	0.1	0.07	0.004
Fat, g/100 g milk	4.93^{BC} (4.79, 5.06)	4.84^{C} (4.71, 4.98)	5.26^{AB} (4.97, 5.54)	5.47^{A} (5.22, 5.72)	0.007	0.3	0.02
Fat, g/milking	634 (606, 662)	662 (633, 690)	670 (611, 729)	613 (560, 665)	0.2	0.1	0.03
MUN, mg/100 g milk	11.55 (11.03, 12.07)	11.45 (10.89, 12.02)	11.50 (10.38, 12.63)	12.14 (11.14, 13.13)	0.7	0.02	-
Fatty acids:							
De novo, g/100 g milk	1.04^{A} (1.01, 1.07)	0.92^{B} (0.89, 0.95)	0.99^{AB} (0.92, 1.06)	0.97^{AB} (0.91, 1.03)	<0.001	-	-
De novo, rel%	22.26^{A} (21.63, 22.90)	20.03^{B} (19.35, 20.72)	19.87^{B} (18.50, 21.23)	18.51^{B} (17.30, 19.72)	<0.001	<0.001	-
De novo, g/milking	130^{A} (125, 135)	126^{AB} (121, 131)	125^{AB} (115, 135)	113^{B} (104, 122)	0.01	0.05	-
Mixed, g/100 g milk	1.50^{AB} (1.45, 1.54)	1.41^{B} (1.36, 1.46)	1.59^{A} (1.49, 1.69)	1.51^{AB} (1.43, 1.60)	0.003	0.002	-
Mixed, rel%	31.95^{A} (31.22, 32.69)	30.39^{B} (29.57, 31.21)	31.63^{AB} (29.98, 33.29)	28.84^{B} (27.38, 30.30)	<0.001	-	-
Mixed, g/milking	188^{AB} (181, 194)	192^{AB} (185, 199)	200^{A} (186, 215)	174^{B} (161, 187)	0.05	0.003	-
Preformed, g/100 g milk	2.18^{B} (2.07, 2.29)	2.31^{B} (2.20, 2.42)	2.46^{AB} (2.23, 2.69)	2.77^{A} (2.56, 2.97)	0.007	0.2	0.04
Preformed, rel%	45.47^{B} (44.24, 46.69)	49.69^{A} (48.31, 51.06)	48.75^{AB} (45.98, 51.52)	52.46^{A} (50.02, 54.90)	<0.001	-	-
Preformed, g/milking	268^{B} (251, 286)	307^{A} (287, 328)	303^{AB} (264, 348)	288^{AB} (254, 325)	0.02	0.2	0.04
Energy related metabolites:							
mpbNEFA, µmol/L	738^{C} (691, 785)	859^{B} (808, 910)	833^{ABC} (731, 934)	1001^{A} (911, 1091)	<0.001	<0.001	-
mBHB, mmol/L	0.08^{B} (0.07, 0.08)	0.09^{A} (0.09, 0.10)	0.10^{AB} (0.08, 0.12)	0.09^{AB} (0.07, 0.10)	0.01	0.5	0.03
mAcetone, mmol/L	0.11^{B} (0.10, 0.13)	0.14^{A} (0.13, 0.16)	0.15^{A} (0.13, 0.18)	0.14^{AB} (0.12, 0.17)	0.003	0.2	0.01

**6 DIM**							
Cows	n= 105	n= 87	n= 23	n= 27			
Milk weight/milking, kg	13.4^{AB} (12.8, 13.9)	14.3^{A} (13.8, 14.9)	11.8^{BC} (10.8, 12.9)	11.9^{C} (10.9, 12.9)	<0.001	0.005	-
Constituents:							
Lactose, g/100 g milk	4.42 (4.39, 4.46)	4.44 (4.40, 4.47)	4.37 (4.31, 4.44)	4.40 (4.34, 4.46)	0.3	0.02	-
Lactose, g/milking	591^{A} (568, 615)	637^{A} (611, 662)	519^{B} (471, 567)	524^{B} (480, 569)	<0.001	0.002	-
Protein, g/100 g milk	3.84^{A} (3.79, 3.90)	3.75^{AB} (3.69, 3.81)	3.61^{B} (3.50, 3.73)	3.66^{B} (3.55, 3.77)	<0.001	-	-
Protein, g/milking	514^{A} (494, 533)	535^{A} (513, 556)	428^{B} (388, 469)	437^{B} (400, 474)	<0.001	0.01	-
Fat, g/100 g milk	4.83 (4.70, 4.96)	4.86 (4.71, 5.00)	5.10 (4.82, 5.38)	5.10 (4.84, 5.36)	0.1	-	-
Fat, g/milking	654 (626, 682)	676 (645, 707)	594 (534, 653)	621 (566, 676)	0.07	-	-
MUN, mg/100 g milk	11.67 (11.22, 12.11)	12.16 (11.67, 12.65)	12.21 (11.25, 13.16)	11.51 (10.62, 12.39)	0.4	-	-
Fatty acids:							
De novo, g/100 g milk	1.01^{A} (0.98, 1.04)	0.90^{B} (0.87, 0.94)	0.87^{B} (0.80, 0.94)	0.92^{AB} (0.86, 0.99)	<0.001	-	-
De novo, rel%	22.06^{A} (21.44, 22.68)	19.55^{B} (18.88, 20.23)	18.10^{B} (16.77, 19.42)	19.15^{B} (17.93, 20.36)	<0.001	-	-
De novo, g/milking	135^{A} (130, 141)	125^{AB} (119, 132)	103^{C} (91, 115)	112^{BC} (101, 123)	<0.001	-	-
Mixed, g/100 g milk	1.49 (1.45, 1.54)	1.46 (1.41, 1.51)	1.53 (1.43, 1.63)	1.49 (1.39, 1.58)	0.6	-	-
Mixed, rel%	32.67 (31.93, 33.41)	31.49 (30.67, 32.30)	31.45 (29.87, 33.04)	30.81 (29.34, 32.27)	0.06	-	-
Mixed, g/milking	201 (193, 209)	202 (193, 211)	178 (160, 196)	180 (164, 196)	0.01	-	-
Preformed, g/100 g milk	2.04^{B} (1.95, 2.13)	2.23^{A} (2.13, 2.34)	2.42^{A} (2.21, 2.65)	2.38^{A} (2.19, 2.59)	<0.001	-	-
Preformed, rel%	45.27^{B} (44.00, 46.54)	48.96^{A} (47.56, 50.36)	50.45^{A} (47.73, 53.17)	50.05^{A} (47.54, 52.55)	<0.001	-	-
Preformed, g/milking	271^{B} (255, 288)	309^{A} (289, 330)	274^{AB} (241, 311)	280^{AB} (249, 316)	0.03	-	-
Energy related metabolites:							
mpbNEFA, µmol/L	715^{B} (669, 762)	839^{A} (788, 890)	873^{A} (773, 972)	865^{A} (773, 957)	<0.001	-	-
mBHB, mmol/L	0.10 (0.09, 0.10)	0.11 (0.10, 0.12)	0.11 (0.09, 0.14)	0.08 (0.06, 0.10)	0.1	0.8	0.03
mAcetone, mmol/L	0.14 (0.12, 0.15)	0.16 (0.15, 0.17)	0.17 (0.14, 0.20)	0.13 (0.10, 0.15)	0.05	0.9	0.008
**7 DIM**							
Cows	n= 89	n= 85	n= 20	n= 25			
Milk weight/milking, kg	14.0^{AB} (13.5, 14.5)	14.7^{A} (14.2, 15.2)	14.1^{AB} (13.1, 15.1)	13.3^{B} (12.4, 14.2)	0.03	-	-
Constituents:							
Lactose, g/100 g milk	4.50 (4.47, 4.52)	4.47 (4.45, 4.50)	4.44 (4.39, 4.50)	4.47 (4.42, 4.52)	0.3	-	-
Lactose, g/milking	629^{AB} (608, 651)	658^{A} (636, 680)	625^{AB} (580, 671)	595^{B} (555, 635)	0.04	-	-
Protein, g/100 g milk	3.60 (3.53, 3.68)	3.58 (3.53, 3.64)	3.60 (3.46, 3.75)	3.68 (3.55, 3.80)	0.007	0.3	0.003
Protein, g/milking	515 (497, 533)	527 (509, 546)	497 (459, 535)	486 (452, 520)	0.1	-	-
Fat, g/100 g milk	4.67^{B} (4.54, 4.80)	4.70^{AB} (4.57, 4.84)	5.08^{A} (4.79, 5.38)	4.88^{AB} (4.63, 5.13)	0.04	-	-
Fat, g/milking	658 (631, 684)	692 (665, 719)	719 (664, 775)	656 (606, 706)	0.1	-	-
MUN, mg/100 g milk	12.07^{A} (11.64, 12.51)	12.29^{A} (11.87, 12.71)	11.43^{AB} (10.57, 12.29)	10.92^{B} (10.15, 11.69)	0.01	0.05	-
Fatty acids:							
De novo, g/100 g milk	0.91 (0.87, 0.96)	0.88 (0.84, 0.91)	0.89 (0.79, 0.98)	0.96 (0.88, 1.04)	0.002	0.5	0.03
De novo, rel%	20.46 (19.60, 21.32)	19.48 (18.79, 20.17)	18.45 (16.70, 20.20)	20.89 (19.40, 22.39)	0.005	0.4	0.002
De novo, g/milking	133 (128, 139)	128 (122, 133)	121 (110, 133)	125 (115, 135)	0.2	-	-
Mixed, g/100 g milk	1.48 (1.43, 1.53)	1.43 (1.39, 1.48)	1.52 (1.42, 1.62)	1.51 (1.42, 1.60)	0.3	-	-
Mixed, rel%	33.28^{A} (32.54, 34.02)	31.66^{B} (30.91, 32.42)	31.32^{AB} (29.76, 32.87)	32.41^{AB} (31.02, 33.81)	0.01	-	-
Mixed, g/milking	206 (198, 214)	208 (200, 217)	213 (196, 230)	200 (186, 215)	0.7	-	-
Preformed, g/100 g milk	1.98^{B} (1.90, 2.08)	2.18^{A} (2.08, 2.29)	2.44^{A} (2.22, 2.69)	2.16^{AB} (1.99, 2.35)	<0.001	-	-

Preformed, rel%	46.94 (45.23, 48.65)	48.79 (47.42, 50.16)	49.76 (46.29, 53.23)	46.38 (43.41, 49.35)	0.02	0.4	0.02
Preformed, g/milking	303 (280, 325)	323 (305, 341)	347 (301, 393)	285 (246, 324)	0.07	0.7	0.01
Energy related metabolites:							
mpbNEFA, µmol/L	720^{B} (671, 770)	813^{A} (765, 860)	871^{A} (773, 969)	786^{AB} (698, 875)	0.02	0.004	-
mBHB, mmol/L	0.10^{B} (0.09, 0.11)	0.12^{A} (0.11, 0.13)	0.13^{AB} (0.11, 0.15)	0.10^{AB} (0.08, 0.12)	0.01	-	-
mAcetone, mmol/L	0.14^{B} (0.13, 0.15)	0.17^{AB} (0.15, 0.18)	0.19^{A} (0.16, 0.21)	0.15^{AB} (0.12, 0.17)	0.005	-	-
**8 DIM**							
Cows	n= 90	n= 83	n= 22	n= 24			
Milk weight/milking, kg	14.2^{B} (13.7, 14.6)	15.2^{A} (14.7, 15.7)	13.5^{B} (12.5, 14.4)	13.6^{B} (12.7, 14.5)	<0.001	-	-
Constituents:							
Lactose, g/100 g milk	4.49 (4.46, 4.52)	4.51 (4.48, 4.54)	4.50 (4.45, 4.55)	4.51 (4.46, 4.56)	0.7	0.003	-
Lactose, g/milking	638^{B} (617, 658)	686^{A} (664, 708)	605^{B} (563, 647)	616^{B} (576, 656)	<0.001	-	-
Protein, g/100 g milk	3.62^{A} (3.57, 3.68)	3.52^{B} (3.46, 3.57)	3.49^{AB} (3.38, 3.60)	3.53^{AB} (3.43, 3.64)	0.03	-	-
Protein, g/milking	511^{AB} (495, 527)	534^{A} (517, 551)	471^{B} (438, 504)	479^{B} (447, 510)	0.001	-	-
Fat, g/100 g milk	4.79^{AB} (4.67, 4.91)	4.63^{B} (4.51, 4.74)	4.78^{AB} (4.56, 5.01)	4.98^{A} (4.76, 5.20)	0.04	0.001	-
Fat, g/milking	664 (638, 691)	706 (678, 734)	652 (598, 706)	668 (617, 720)	0.1	-	-
MUN, mg/100 g milk	12.53^{A} (12.11, 12.95)	12.26^{A} (11.82, 12.70)	11.82^{AB} (10.97, 12.67)	10.92^{B} (10.10, 11.73)	0.006	-	-
Fatty acids:							
De novo, g/100 g milk	0.97^{A} (0.93, 1.00)	0.88^{B} (0.84, 0.91)	0.87^{B} (0.80, 0.93)	0.91^{AB} (0.85, 0.97)	0.001	0.03	-
De novo, rel%	21.43^{A} (20.73, 22.12)	19.91^{B} (19.23, 20.58)	19.14^{B} (17.83, 20.45)	19.35^{B} (18.07, 20.62)	<0.001	0.04	-
De novo, g/milking	132 (126, 139)	132 (127, 138)	120 (108, 131)	121 (107, 134)	0.4	0.1	0.02
Mixed, g/100 g milk	1.50 (1.46, 1.54)	1.43 (1.38, 1.47)	1.48 (1.40, 1.57)	1.52 (1.44, 1.60)	0.06	-	-
Mixed, rel%	33.79^{A} (33.05, 34.54)	32.21^{B} (31.43, 32.99)	32.43^{AB} (30.91, 33.95)	32.69^{AB} (31.24, 34.14)	0.03	-	-
Mixed, g/milking	211 (203, 219)	216 (208, 224)	200 (184, 216)	206 (191, 221)	0.3	-	-
Preformed, g/100 g milk	2.07 (1.97, 2.17)	2.12 (2.02, 2.21)	2.21 (2.02, 2.40)	2.31 (2.13, 2.50)	0.09	0.001	-
Preformed, rel%	44.54^{B} (43.23, 45.85)	47.87^{A} (46.50, 49.23)	48.58^{A} (45.93, 51.22)	47.78^{AB} (45.25, 50.32)	0.001	-	-
Preformed, g/milking	294 (275, 314)	324 (305, 343)	299 (263, 336)	317 (282, 352)	0.2	0.04	-
Energy related metabolites:							
mpbNEFA, µmol/L	695 (647, 744)	770 (723, 817)	775 (684, 866)	783 (695, 871)	0.09	<0.001	-
mBHB, mmol/L	0.11 (0.10, 0.11)	0.12 (0.11, 0.13)	0.12 (0.10, 0.14)	0.11 (0.09, 0.13)	0.09	-	-
mAcetone, mmol/L	0.15 (0.13, 0.16)	0.17 (0.15, 0.18)	0.17 (0.15, 0.20)	0.15 (0.13, 0.17)	0.07	-	-
**9 DIM**							
Cows	n= 93	n= 85	n= 12	n= 27			
Milk weight/milking, kg	14.3^{B} (13.9, 14.8)	15.4^{A} (14.9, 15.9)	14.3^{AB} (13.0, 15.6)	13.2^{B} (12.3, 14.1)	<0.001	0.02	-
Constituents:							
Lactose, g/100 g milk	4.52 (4.50, 4.55)	4.53 (4.51, 4.56)	4.51 (4.44, 4.58)	4.53 (4.48, 4.58)	0.9	<0.001	-
Lactose, g/milking	649^{B} (626, 672)	697^{A} (674, 719)	646^{AB} (587, 706)	600^{B} (560, 640)	<0.001	0.005	-
Protein, g/100 g milk	3.58^{A} (3.52, 3.63)	3.42^{B} (3.36, 3.48)	3.41^{AB} (3.26, 3.56)	3.39^{B} (3.29, 3.49)	<0.001	-	-
Protein, g/milking	510^{A} (493, 526)	525^{A} (509, 541)	491^{AB} (447, 534)	446^{B} (417, 475)	<0.001	0.02	-
Fat, g/100 g milk	4.66 (4.55, 4.78)	4.67 (4.56, 4.79)	4.76 (4.46, 5.06)	4.59 (4.38, 4.79)	0.8	0.02	-
Fat, g/milking	666^{B} (641, 690)	713^{A} (687, 739)	686^{AB} (618, 755)	610^{B} (564, 656)	0.001	-	-
MUN, mg/100 g milk	12.07^{A} (11.60, 12.54)	12.37^{A} (11.90, 12.83)	11.96^{AB} (10.72, 13.20)	10.43^{B} (9.60, 11.26)	0.001	0.004	-
Fatty acids:							
De novo, g/100 g milk	0.96^{A} (0.93, 0.99)	0.86^{B} (0.83, 0.90)	0.90^{AB} (0.81, 0.99)	0.85^{B} (0.79, 0.91)	<0.001	-	-

De novo, rel%	21.98^{A} (21.34, 22.62)	19.40^{B} (18.72, 20.07)	19.97^{AB} (18.18, 21.76)	19.78^{B} (18.59, 20.97)	<0.001	-	-
De novo, g/milking	137^{A} (132, 142)	131^{A} (126, 136)	130^{AB} (116, 144)	114^{B} (104, 123)	<0.001	-	-
Mixed, g/100 g milk	1.49 (1.45, 1.53)	1.43 (1.38, 1.47)	1.47 (1.36, 1.59)	1.42 (1.35, 1.50)	0.1	-	-
Mixed, rel%	33.68^{A} (32.90, 34.47)	31.99^{B} (31.27, 32.71)	32.57^{AB} (30.50, 34.65)	33.39^{AB} (31.98, 34.81)	0.03	0.1	0.02
Mixed, g/milking	214^{A} (207, 221)	217^{A} (209, 224)	212^{AB} (192, 232)	190^{B} (176, 203)	0.007	-	-
Preformed, g/100 g milk	1.99^{B} (1.89, 2.09)	2.18^{A} (2.09, 2.27)	2.17^{AB} (1.91, 2.43)	2.03^{AB} (1.85, 2.20)	0.3	0.2	0.05
Preformed, rel%	43.85^{B} (42.60, 45.09)	48.65^{A} (47.35, 49.95)	47.42^{AB} (43.96, 50.88)	47.34^{A} (45.03, 49.65)	<0.001	-	-
Preformed, g/milking	281^{B} (264, 298)	333^{A} (315, 350)	313^{AB} (266, 359)	280^{B} (249, 311)	<0.001	-	-
Energy related metabolites:							
mpbNEFA, µmol/L	652^{B} (608, 696)	788^{A} (744, 832)	708^{AB} (591, 824)	693^{AB} (615, 771)	<0.001	0.02	-
mBHB, mmol/L	0.11^{B} (0.10, 0.12)	0.13^{A} (0.12, 0.14)	0.12^{AB} (0.10, 0.15)	0.09^{B} (0.07, 0.11)	0.02	0.08	0.02
mAcetone, mmol/L	0.15^{B} (0.13, 0.16)	0.18^{A} (0.16, 0.19)	0.16^{AB} (0.12, 0.19)	0.12^{B} (0.10, 0.15)	0.03	0.2	0.03
**10 DIM**							
Cows	n= 96	n= 76	n= 11	n= 26			
Milk weight/milking, kg	15.0^{A} (14.5, 15.5)	15.6^{A} (15.0, 16.1)	14.8^{AB} (13.4, 16.2)	13.3^{B} (12.4, 14.3)	0.001	0.01	-
Constituents:							
Lactose, g/100 g milk	4.59 (4.56, 4.62)	4.55 (4.52, 4.58)	4.52 (4.43, 4.61)	4.56 (4.50, 4.62)	0.2	-	-
Lactose, g/milking	686^{A} (662, 710)	707^{A} (682, 733)	670^{AB} (604, 737)	608^{B} (565, 651)	0.002	0.006	-
Protein, g/100 g milk	3.45^{A} (3.40, 3.50)	3.36^{AB} (3.30, 3.42)	3.21^{B} (3.05, 3.36)	3.34^{AB} (3.24, 3.44)	0.008	-	-
Protein, g/milking	514^{A} (497, 532)	523^{A} (504, 541)	472^{AB} (423, 521)	446^{B} (414, 477)	<0.001	0.01	-
Fat, g/100 g milk	4.61 (4.48, 4.74)	4.58 (4.44, 4.73)	4.69 (4.31, 5.06)	4.54 (4.29, 4.79)	0.9	-	-
Fat, g/milking	694^{A} (666, 721)	707^{A} (678, 737)	699^{AB} (622, 777)	607^{B} (557, 658)	0.008	0.03	-
MUN, mg/100 g milk	12.11 (11.68, 12.54)	12.00 (11.54, 12.45)	11.63 (10.43, 12.84)	11.53 (10.75, 12.31)	0.6	0.003	-
Fatty acids:							
De novo, g/100 g milk	0.94^{A} (0.91, 0.97)	0.86^{B} (0.82, 0.89)	0.88^{AB} (0.78, 0.97)	0.90^{AB} (0.84, 0.96)	0.01	-	-
De novo, rel%	21.21^{A} (20.56, 21.87)	19.75^{B} (19.11, 20.40)	20.71^{AB} (18.83, 22.58)	21.00^{AB} (19.87, 22.14)	0.09	0.2	0.04
De novo, g/milking	139^{A} (134, 145)	133^{AB} (127, 139)	128^{AB} (112, 145)	119^{B} (108, 130)	0.008	-	-
Mixed, g/100 g milk	1.48 (1.43, 1.52)	1.40 (1.36, 1.45)	1.49 (1.37, 1.61)	1.46 (1.38, 1.54)	0.1	-	-
Mixed, rel%	33.28^{AB} (32.55, 34.01)	32.33^{B} (31.60, 33.05)	35.39^{A} (33.29, 37.49)	33.92^{AB} (32.65, 35.20)	0.02	0.01	0.02
Mixed, g/milking	218^{A} (210, 227)	218^{A} (209, 227)	221^{AB} (197, 244)	193^{B} (178, 208)	0.03	0.03	-
Preformed, g/100 g milk	2.01 (1.91, 2.11)	2.11 (2.01, 2.21)	1.92 (1.62, 2.21)	1.96 (1.78, 2.14)	0.6	0.008	0.04
Preformed, rel%	45.51 (44.24, 46.78)	47.92 (46.67, 49.17)	43.90 (40.27, 47.54)	45.08 (42.87, 47.28)	0.04	0.03	0.01
Preformed, g/milking	302^{AB} (285, 319)	324^{A} (306, 342)	317^{AB} (269, 365)	267^{B} (236, 298)	0.02	0.007	-
Energy related metabolites:							
mpbNEFA, µmol/L	682 (637, 728)	756 (708, 804)	668 (540, 795)	646 (563, 729)	0.06	<0.001	-
mBHB, mmol/L	0.12^{A} (0.11, 0.13)	0.13^{A} (0.12, 0.14)	0.10^{AB} (0.07, 0.13)	0.09^{B} (0.07, 0.11)	0.2	0.03	0.02
mAcetone, mmol/L	0.15^{AB} (0.14, 0.17)	0.17^{A} (0.16, 0.19)	0.13^{AB} (0.10, 0.17)	0.12^{B} (0.10, 0.14)	0.2	0.02	0.004

[1] MUN = Milk urea nitrogen
[2] rel% = relative percentage of total fatty acid g/100 g milk
[3] mpbNEFA = milk predicted blood non-esterified fatty acid
[4] mBHB = milk β-hydroxybutyrate
^{ABC} Letters differing amongst a row indicate statistically significant differences between groups based on Tukey corrected $P < 0.05$.
