# Supplementary material for: Calcium dynamics and associated temporal patterns of milk constituents in early-lactation multiparous Holsteins
Source: J Dairy Sci. Author manuscript; Available in PMC 2025 Dec 15. (PMC12703615; doi:10.3168/jds.2022-23142)
Supplement: Supplemental Table 3 [file NIHMS2111481-supplement-Supplemental_Table_3.pdf]

Supplemental Table 3. Means and 95% confidence intervals of modeled milk estimates of 343 multiparous Holsteins on a commercial dairy in Cayuga County, NY, sampled from 3 to 10 DIM by Ca dynamic group. Calcium dynamic group classification was based on subclinical hypocalcemia (**SCH**) status at 1 DIM (tCa < 1.98 mmol/L) and 4 DIM (tCa < 2.22 mmol/L). Groups defined as normocalcemic (**NC**) and cows experiencing transient (**tSCH**; SCH at 1 DIM only), persistent (**pSCH**; SCH at 1 and 4 DIM), and delayed SCH (**dSCH**; SCH at 4 DIM only).

	Calcium dynamic group				*P*-values	
	NC	tSCH	pSCH	dSCH	Group	Parity
**All DIM**						
Sample size	n= 733	n= 643	n= 140	n= 201		
Milk weight/milking, kg	13.4^{B} (13.0, 13.8)	14.4^{A} (14.1, 14.7)	13.1^{BC} (12.3, 13.8)	12.2^{C} (11.5, 12.8)	<0.001	0.005
Constituents:						
Lactose, g/100 g milk	4.44 (4.42, 4.45)	4.45 (4.43, 4.46)	4.39 (4.34, 4.45)	4.39 (4.35, 4.44)	0.07	<0.001
Lactose, g/milking	595^{B} (578, 611)	640^{A} (626, 655)	576^{BC} (543, 609)	537^{C} (507, 567)	<0.001	<0.001
Protein, g/100 g milk	3.81^{A} (3.77, 3.85)	3.72^{B} (3.67, 3.76)	3.64^{B} (3.55, 3.73)	3.66^{AB} (3.55, 3.76)	<0.001	-
Protein, g/milking	506^{B} (494, 518)	530^{A} (518, 541)	474^{BC} (444, 504)	442^{C} (417, 468)	<0.001	0.007
Fat, g/100 g milk	4.85^{AB} (4.75, 4.95)	4.80^{B} (4.70, 4.90)	5.09^{A} (4.91, 5.26)	5.11^{A} (4.92, 5.31)	0.003	<0.001
Fat, g/milking	647^{AB} (624, 669)	683^{A} (667, 700)	664^{AB} (613, 714)	615^{B} (576, 655)	0.007	0.05
MUN[1], mg/100 g milk	11.51 (11.18, 11.84)	11.50 (11.16, 11.83)	11.42 (10.76, 12.08)	11.34 (10.69, 11.99)	0.9	-
Fatty acids:						
De novo, g/100 g milk	1.01^{A} (0.98, 1.03)	0.92^{B} (0.90, 0.94)	0.93^{B} (0.88, 0.97)	0.95^{AB} (0.90, 1.00)	<0.001	-
De novo, rel%[2]	21.96^{A} (21.42, 22.51)	20.02^{B} (19.58, 20.47)	19.24^{B} (18.16, 20.31)	19.67^{B} (18.48, 20.87)	<0.001	0.003
De novo, g/milking	134^{A} (131, 138)	129^{AB} (126, 133)	120^{BC} (111, 130)	115^{C} (107, 123)	<0.001	-
Mixed, g/100 g milk	1.48^{AB} (1.44, 1.51)	1.42^{B} (1.38, 1.45)	1.52^{A} (1.47, 1.58)	1.51^{AB} (1.44, 1.57)	<0.001	-
Mixed, rel%	32.52^{A} (31.92, 33.12)	30.89^{B} (30.35, 31.43)	31.31^{AB} (29.98, 32.64)	31.43^{AB} (30.10, 32.76)	<0.001	-
Mixed, g/milking	197^{AB} (192, 203)	202^{A} (197, 207)	198^{AB} (185, 210)	182^{B} (171, 193)	0.02	0.04
Preformed, g/100 g milk	2.13^{B} (2.05, 2.21)	2.27^{AB} (2.19, 2.34)	2.43^{A} (2.25, 2.61)	2.44^{A} (2.25, 2.63)	<0.001	<0.001
Preformed, rel%	45.29^{B} (44.27, 46.31)	49.10^{A} (48.21, 49.98)	49.63^{A} (47.31, 51.96)	48.74^{AB} (46.38, 51.10)	<0.001	-
Preformed, g/milking	286^{B} (271, 302)	323^{A} (311, 334)	317^{AB} (283, 352)	293^{AB} (265, 322)	0.005	0.02
Energy related metabolites:						
mpbNEFA[3], µmol/L	717^{B} (678, 755)	821^{A} (787, 854)	820^{AB} (736, 904)	826^{AB} (741, 912)	<0.001	<0.001
mBHB[4], mmol/L	0.09^{B} (0.08, 0.09)	0.11^{A} (0.10, 0.11)	0.10^{AB} (0.09, 0.12)	0.09^{AB} (0.07, 0.10)	<0.001	-
mAcetone, mmol/L	0.13^{B} (0.12, 0.14)	0.15^{A} (0.14, 0.16)	0.16^{AB} (0.13, 0.18)	0.14^{AB} (0.12, 0.16)	<0.001	0.02

[1] MUN = Milk urea nitrogen
[2] rel% = relative percentage of total fatty acid g/100 g milk
[3] mpbNEFA = milk predicted blood non-esterified fatty acid
[4] mBHB = milk β-hydroxybutyrate
^{ABC} Letters differing amongst a row indicate statistically significant differences between groups at based on Bonferroni corrected $P < 0.05$.
